# Global trends of interstitial lung diseases from 1990 to 2019: an age–period–cohort study based on the Global Burden of Disease study 2019, and projections until 2030

**DOI:** 10.3389/fmed.2023.1141372

**Published:** 2023-07-24

**Authors:** Qi Zeng, Depeng Jiang

**Affiliations:** Department of Respiratory Medicine, Second Affiliated Hospital of Chongqing Medical University, Chongqing, China

**Keywords:** interstitial lung disease, pneumoconiosis, Global Burden of Disease (GBD), age–period–cohort (APC) analysis, prevalence, mortality, projection

## Abstract

**Background:**

Interstitial lung diseases (ILDs) are indispensable components of chronic respiratory diseases and global health challenges. We aimed to explore the global long-term changes in the prevalence, mortality, and disability-adjusted life years (DALYs) of ILDs; investigate the independent effect of age, period, and cohort; and project the disease burden over the next decade.

**Methods:**

Data were retrieved from the Global Burden of Disease (GBD) database 2019. The joinpoint regression model was used to calculate the average annual percent change (AAPC). An age–period–cohort (APC) analysis was employed to measure the independent effect of age, period, and cohort. The Bayesian age–period–cohort (BAPC) model was used to project the global epidemiological trends until 2030.

**Results:**

From 1990 to 2019, the age-standardized prevalence rate (ASPR), age-standardized mortality rate (ASMR), and age-standardized disability-adjusted life years (DALYs) rate (ASDR) of interstitial lung disease and pulmonary sarcoidosis (ILD) slightly increased from 52.66 per 100,000 [95% uncertainty interval (UI) 44.49 to 61.07] to 57.62 per 100,000 (95% UI 49.42 to 65.67), from 1.76 per 100,000 (95% UI 1.41 to 2.22) to 2.17 per 100,000 (95% UI 1.5 to 2.62), and from 41.57 per 100,000 (95% UI 33.93 to 51.92) to 46.45 per 100,000 (95% UI 35.12 to 54.98), whereas the ASPR, ASMR, and ASDR of pneumoconiosis decreased. High social-demographic index (SDI) regions possessed the highest ASPR, whereas low-middle SDI regions had the highest ASMR and ASDR, followed by low-SDI regions in ILD. Middle-SDI regions reported the highest ASPR, ASMR, and ASDR in pneumoconiosis. The age effect showed that the rate ratio (RR) was high in older adults. Period effect indicated that the RR of prevalence increased over time, whereas the RR of mortality and DALYs decreased in men but increased in women. The cohort effect exhibited that the more recent birth cohort had a higher RR than the previous cohort in prevalence. We projected that ASPR, ASMR, and ASDR would stabilize with little variation over the next decade.

**Conclusion:**

The global burden of ILDs remains relatively severe, especially among older adults, in low- and middle-SDI regions. Effective measurements are expected to improve this situation.

## 1. Introduction

Interstitial lung diseases (ILDs) cover more than 200 diseases, characterized by inflammation or fibrosis within the pulmonary mesenchyme, alveolar wall, and alveolar space, defined by their causes and distinct histopathological features, from extremely rare to relatively common (1, 2). Sarcoidosis is a granulomatous disease that can affect virtually any organ, in which pulmonary involvement is highly prevalent (3). Fibrosis has frequently been observed in sarcoidosis, and as a confounding histological feature, other conditions such as fibrotic ILDs and pneumoconiosis (particularly when associated with dust) are also associated with this finding (3). The pulmonary manifestations of sarcoidosis vary significantly, ranging from asymptomatic to life-threatening cases, with pulmonary fibrosis accounting for most cases of mortality associated with the disease (4). Identifiable causes of ILDs include occupational exposure, drugs or radiation therapy, and connective tissue disease (CTD-ILD) (5). Patients with unknown causes are defined as those with idiopathic interstitial pneumonia, of which idiopathic pulmonary fibrosis (IPF) is the most common and lethal type, associated with gene polymorphisms related to host defense and cell repair (6, 7).

ILDs can cause irreversible lung damage, resulting in an impaired quality of life, permanent physical disabilities, and respiratory failure, imposing a significant burden on society. One of the first published ILDs registries in New Mexico suggested that the prevalence of ILDs in the general population may be greater than previously estimated and that 90% of the reported occupational diseases in China were pneumoconiosis (8, 9). Additionally, ILDs are considered the primary cause of death in patients with systemic sclerosis, and the mortality rate of IPF has increased by 9.85% from 2000 to 2017 in the United States (10, 11). Moreover, with CTD-ILD, people experience a lower employment rate and workplace productivity loss estimated at $13,593 per person; the mean total 5-year cost for rheumatoid arthritis patients with ILDs was US $173,405 (12, 13).

The Global Alliance against Chronic Respiratory Diseases (GARD) is devoted to initiating a comprehensive approach to fight chronic respiratory diseases, with the vision of a world where all people breathe freely (14). Understanding the informative epidemiological estimates of ILDs in specific regions and populations enables us to formulate better health-related policies, facilitate the reasonable allocation of healthcare resources, and alleviate the burden of ILDs. Nevertheless, comprehensive data on ILDs on a global scale are scarce. Previous studies have mainly analyzed specific types of ILDs and were limited to localized regions (15–17). In this study, the results of the updated Global Burden of Disease (GBD) 2019 on ILDs are presented. We visualized the prevalence, mortality, and disability-adjusted life years (DALYs) data of ILDs from the GBD2019 by age, sex, year, and region; analyzed the age, period, and cohort effects on the changes in prevalence, mortality, and DALYs; and projected the overall trends of ILD until 2030.

## 2. Methods

### 2.1. Data source

In the International Classification of Diseases, 10th edition (ICD-10), the codes of “other respiratory diseases principally affecting the interstitium” were J80–J84, among which J84 was the code for “other interstitial pulmonary diseases,” the code of “sarcoidosis” was D86 and that of “pulmonary sarcoidosis” was D86.001, and the codes of “lung diseases due to external agents” were J60–J70, among which J60 was the code for “coal worker's pneumoconiosis” and J61 was the code for “pneumoconiosis due to asbestos and other mineral fibers.” In the GBD study, interstitial lung diseases (ILDs) and pulmonary sarcoidosis are defined as a collection of chronic respiratory diseases that impair lung function and oxygen uptake through scarring and/or inflammation. Pneumoconiosis is defined as a chronic lung disease typified by lung scarring and other interstitial damage caused by exposure to dust and other containments—usually through occupational exposure. The relevant ICD codes for ILD are J84 and D86, the American Thoracic Society was used as the gold-standard definition for ILD, and exposure types such as coal, asbestos, and silica were used to model pneumoconiosis (18).

Data on global ILDs prevalence, mortality, and DALYs from 1990 to 2019 were retrieved from the GBD2019 study, which covers 204 countries and territories, providing comprehensive and standardized estimations of 369 diseases and injuries and 87 risk factors, engaging a large network of individual collaborators with specialties in various topic areas. The data source for the GBD study includes censuses, household surveys, civil registration and vital statistics, disease registries, health service use, air pollution monitors, satellite imaging, disease notifications, and other sources. The cause of death database is composed of vital registration, verbal autopsy, registry, survey, police, and surveillance data. Data sources using alternative case definitions or measurement methods were adjusted using network meta-regression to the reference definition or measurement method to be comparable. To standardize the cause of death data, which is organized in a hierarchical list containing four levels, protocols were used to address differences in ICD codes due to national variation or revision. There are three main standardized tools in the GBD study, including the Cause of Death Ensemble model, a highly systematized tool to analyze the cause of death data; spatiotemporal Gaussian process regression, a set of regression methods that borrow strength between locations and over time for single metrics; and DisMod-MR, a Bayesian meta-regression tool that allows evaluation of all available data on prevalence, mortality, and remission for a disease. More details on methodological information and modeling strategies in the GBD study were published elsewhere as Supplementary material (18–20).

The prevalence data for ILDs were primarily derived from hospital inpatient and insurance claims data. Mortality data were derived from vital registration systems, censuses, and surveys. The verbal autopsy was excluded from the model as the sensitivity of verbal autopsy algorithms to detect specific chronic respiratory diseases is poor (18). DALYs are the sum of years lived with disability (YLDs) and years of life lost (YLLs). YLD was calculated by multiplying the prevalence of each sequela, and YLL was calculated by multiplying the number of deaths. Age-standardized rates were based on the GBD global reference population. Social-demographic index (SDI) represents a population's social and economic development status for each location year; a higher index suggests a more developed society. We downloaded the data from the GBD query tools (https://vizhub.healthdata.org/gbd-results/), and GraphPad Prism 9.4.1 and R 4.2.1 were utilized to visualize the data.

### 2.2. Joinpoint regression analysis

Trends in the age-standardized prevalence (ASPR), mortality (ASMR), and DALYs rate (ASDR; per 100,000) of ILDs were evaluated using Joinpoint Software 4.9.1, and the data from GBD were processed in Excel 2019 and R 4.2.1. The modeling strategy of the Joinpoint software was the grid search method, which was used to establish the possible connection nodes (Joinpoint nodes) and calculate the sum of squares errors and mean squared errors in each situation. Five points was the maximum number allowed in our study. The internal trends of each independent interval were evaluated using the annual percent change (APC) and 95% confidence interval (CI), and a comprehensive evaluation of the overall trend was performed using the average APC (AAPC) and 95% CI. Each *p*-value was determined using the Monte Carlo method, and a *p*-value of < 0.05 was considered statistically significant (21).

### 2.3. Age–period–cohort analysis

The APC Web Tool (https://analysistools.cancer.gov/apc/) and R 4.2.1 were employed for data processing (22). We separated the ASPR, ASMR, and ASDR of ILD into consecutive 5-year age intervals, ranging from 0–4 to 95–99 years, and the period from 1990 to 2019 into successive 5-year segments, as it was stipulated in the APC framework that the age and period intervals must be identical. Timepoint values (1992, 1997, …, 2017) were used to replace the 5-year periods to avoid a temporal overlap of the adjacent birth cohorts from 1990 to 2019. The age group of 40–44 years, period 2000–2004, and birth cohort 1955–1959 were selected as the references for age, period, and cohort effect, respectively. The rate ratio (RR) indicates the value of a particular age, period, or cohort compared with the reference value. RR > 1 suggests that the factor has a higher risk of disease than the reference. Local drifts indicate APCs, whereas net drifts indicate the overall APCs. Wald's chi-square tests were used to estimate the significance of parameters and functions. All statistical tests were two-tailed.

### 2.4. Projection of model development

The Bayesian age–period–cohort (BAPC) model was used to conduct the projections. This model has been verified to be superior to many other linear power models and achieves more sensible projections. One study suggested that the probabilistic forecasts obtained using the BAPC model were well-calibrated and not too wide (23). Based on the integrated nested Laplace approximations, the BAPC model assumed that the effect of age, period, and cohort adjacent in time was analogous. The classifications of age groups and periods were the same as those in the APC model. We conducted the BAPC analysis using the R package “BAPC.”

### 2.5. Ethics statement

No animal studies, no human studies, and no potentially identifiable human images or data are presented in this study.

## 3. Results

### 3.1. Global, regional, and national trends in the prevalence, mortality, and DALYs of ILDs

**Figure 1:**
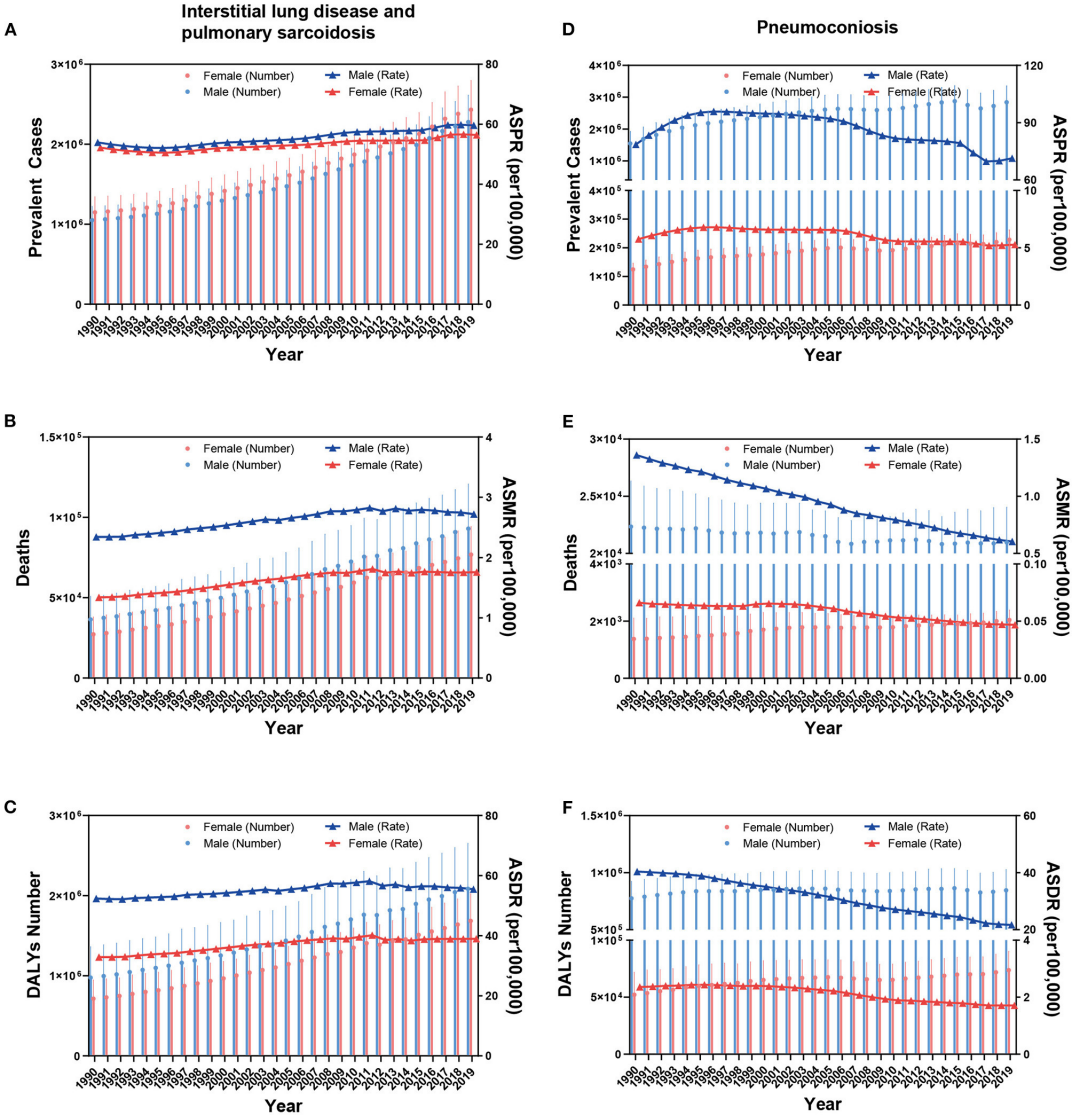
Global prevalent cases, ASPR **(A, D)**, deaths, ASMR **(B, E)**, DALYs number, and ASDR **(C, F)** of ILDs by sex from 1990 to 2019.

According to GBD 2019, an estimated 2.28 million [95% uncertainty interval (UI) 1.95 to 2.61] men and 2.43 million (95% UI 2.07 to 2.8) women worldwide had ILD in 2019 (Figure 1A). From 1990 to 2019, ASMR slightly increased from 2.34 per 100,000 (95% UI 1.6 to 3.18) to 2.72 per 100,000 (95% UI 1.66 to 3.55) in men and from 1.34 per 100,000 (95% UI 1.02 to 1.87) to 1.76 per 100,000 (95% UI 1.11 to 2.16) in women (Figure 1B). ASDR increased from 52.38 per 100,000 (95% UI 37.18 to 71.76) to 55.47 per 100,000 (95% UI 36.9 to 70.32) in men and from 32.86 per 100,000 (95% UI 25.81 to 43.75) to 38.99 per 100,000 (95% UI 28.09 to 46.8) in women, with the annual rate of change of 0.06 (95% UI −0.2 to 0.45) and 0.19 (95% UI −0.06 to 0.43), respectively (Figure 1C). Regarding pneumoconiosis, all the cases and age-standardized rates in men were much greater than those in women. The ASPR of pneumoconiosis in men increased from 78.54 per 100,000 (95% UI 65.35 to 96.72) in 1990 to 95.82 per 100,000 (95% UI 81.99 to 111.95) in 1996 and then gradually decreased to 69.7 per 100,000 (95% UI 58.6 to 81.77) in 2017 and increased to 71.36 (95% UI 60.05 to 83.86) in 2019 (Figure 1D). Both the ASMR and ASDR of pneumoconiosis in men decreased in the last three decades, with the annual rate of change of −0.56 (95% UI −0.63 to −0.41) and −0.46 (95% UI −0.55 to −0.33), respectively. However, number of deaths and DALYs due to pneumoconiosis remained almost unchanged between 1990 and 2019 (Figures 1E, F).

**Figure 2:**
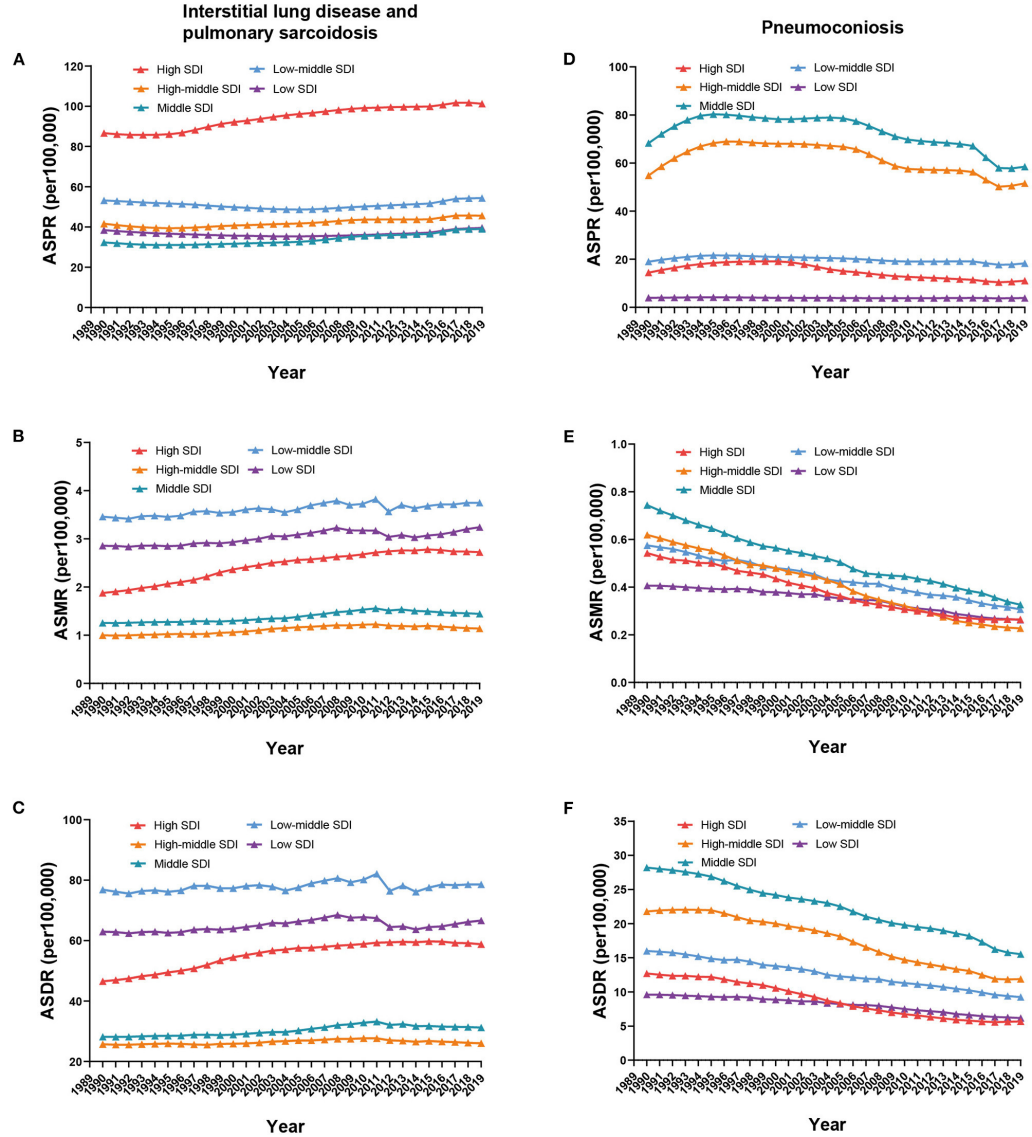
Global ASPR **(A, D)**, ASMR **(B, E)**, and ASDR **(C, F)** of ILDs by SDI regions from 1990 to 2019.

The ASPR of ILD was the highest in high-SDI regions, at 101.21 per 100,000 (95% UI 87.66 to 113.79) in 2019, and a slight upward trend was observed in high-SDI regions from 1990 to 2019, with an annual rate of change of 0.17 (95% UI 0.11 to 0.22); little difference was presented in other SDI regions (Figure 2A). Analogous trends were observed for ASMR and ASDR. The low-middle SDI regions possessed the highest ASMR and ASDR, whereas the high-middle SDI regions had the lowest, at 3.75 per 100,000 (95% UI 2.51 to 5.15) and 1.14 per 100,000 (95% UI 0.81 to 1.34) in ASMR and 78.56 per 100,000 (95% UI 54.31 to 107.04) and 26 per 100,000 (95% UI 20.53 to 30.04) in ASDR in 2019. In high-SDI regions, mild uptrends were exhibited; however, in other SDI regions, the trends almost leveled off from 1990 to 2019 (Figures 2B, C). As for the ASPR of pneumoconiosis, the middle-SDI regions had the maximal rate, at 58.47 per 100,000 (95% UI 48.81 to 69.34) in 2019, followed by the high-middle SDI regions (Figure 2D). The ASMR and ASDR in all SDI regions decreased from 1990 to 2019, and the middle-SDI regions possessed the highest rate, from 0.74 per 100,000 (95% UI 0.52 to 0.94) in 1990 to 0.33 per 100,000 (95% UI 0.27 to 0.4) in 2019 in ASMR and from 28.21 per 100,000 (95% UI 21 to 34.99) in 1990 to 15.52 per 100,000 (95% UI 12.39 to 19.44) in 2019 in ASDR; the low- and high-SDI regions had relatively low rates, with 0.26 per 100,000 (95% UI 0.12 to 0.37) and 0.26 per 100,000 (95% UI 0.23 to 0.3) in ASMR and 6.17 per 100,000 (95% UI 3.09 to 8.6) and 5.67 per 100,000 (95% UI 5 to 6.44) in ASDR in 2019 (Figures 2E, F).

**Figure 3:**
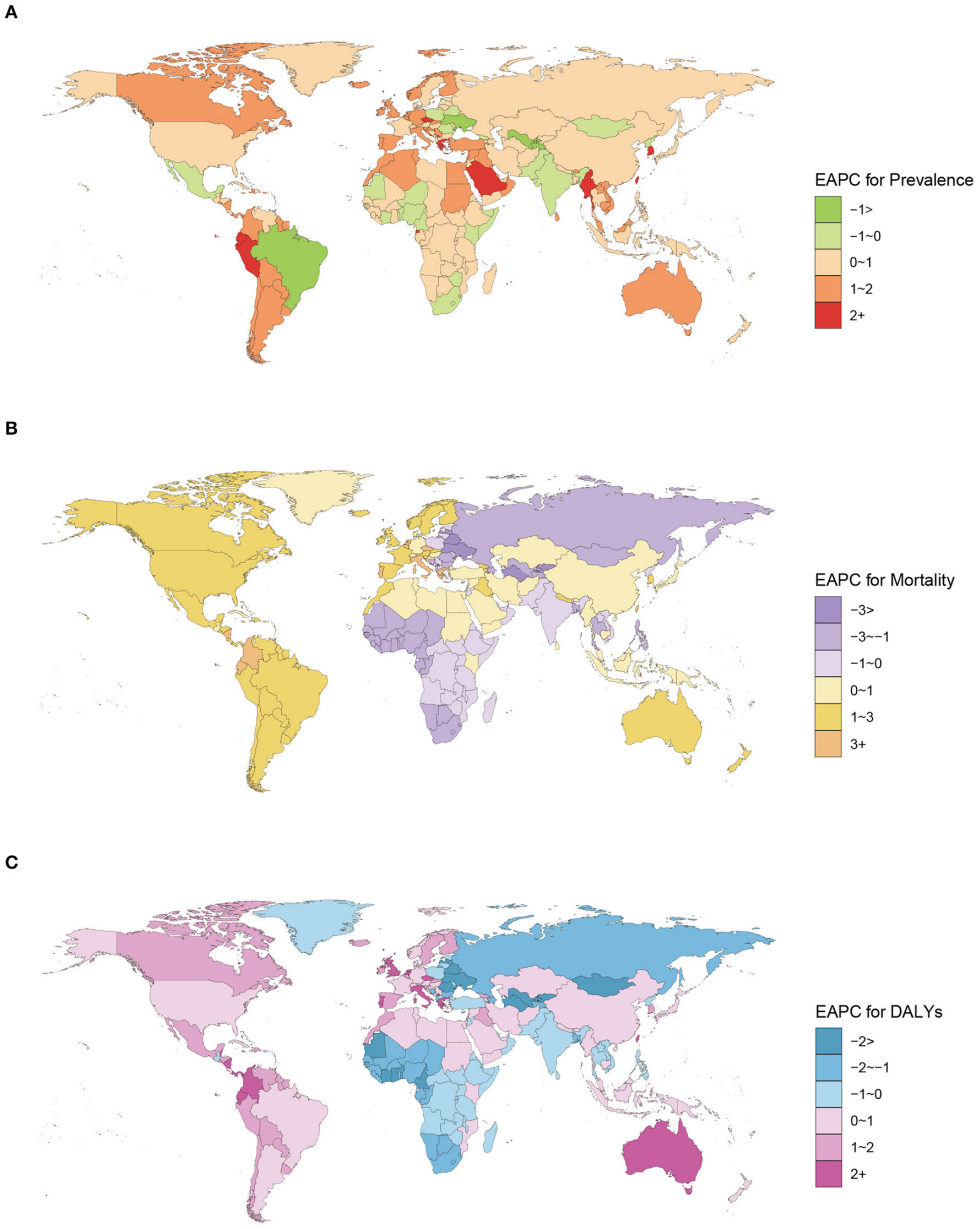
EAPC for prevalence **(A)**, mortality **(B)**, and DALYs **(C)** of ILD in 204 countries and territories from 1990 to 2019.

The ASPR, ASMR, and ASDR of ILD and the SDI value in 204 countries and territories in 1990 and 2019 were determined. In 1990, Brunei, Japan, and the United States ranked as the top three countries with the highest ASPR. Maldives had the highest ASMR and ASDR, followed by Peru (Supplementary Table 1). In 2019, the areas with a higher ASPR were Japan, Peru, Chile, Brunei, the United States, Greenland, Palau, and Maldives, all with over 120 per 100,000. Bolivia had the maximal ASMR, at 11.53 per 100,000 (95% UI 8.33 to 15.48), followed by Peru. ASDR was the highest in Nepal, Bolivia, and Peru and exceeded 190 per 100,0000 (Supplementary Table 2). From 1990 to 2019, ASPR increased in most countries and territories, with higher estimated APC (EAPC) values scattered worldwide (Figure 3A). Most African and South Asian countries and territories had EAPC values of ASMR and ASDR below 0, whereas in North America, South America, and Oceania, the majority of EAPC values were positive (Figures 3B, C). The EAPCs of ASPR, ASMR, and ASDR of pneumoconiosis are shown in Supplementary Figure 1.

### 3.2. Joinpoint regression analysis

**Figure 4:**
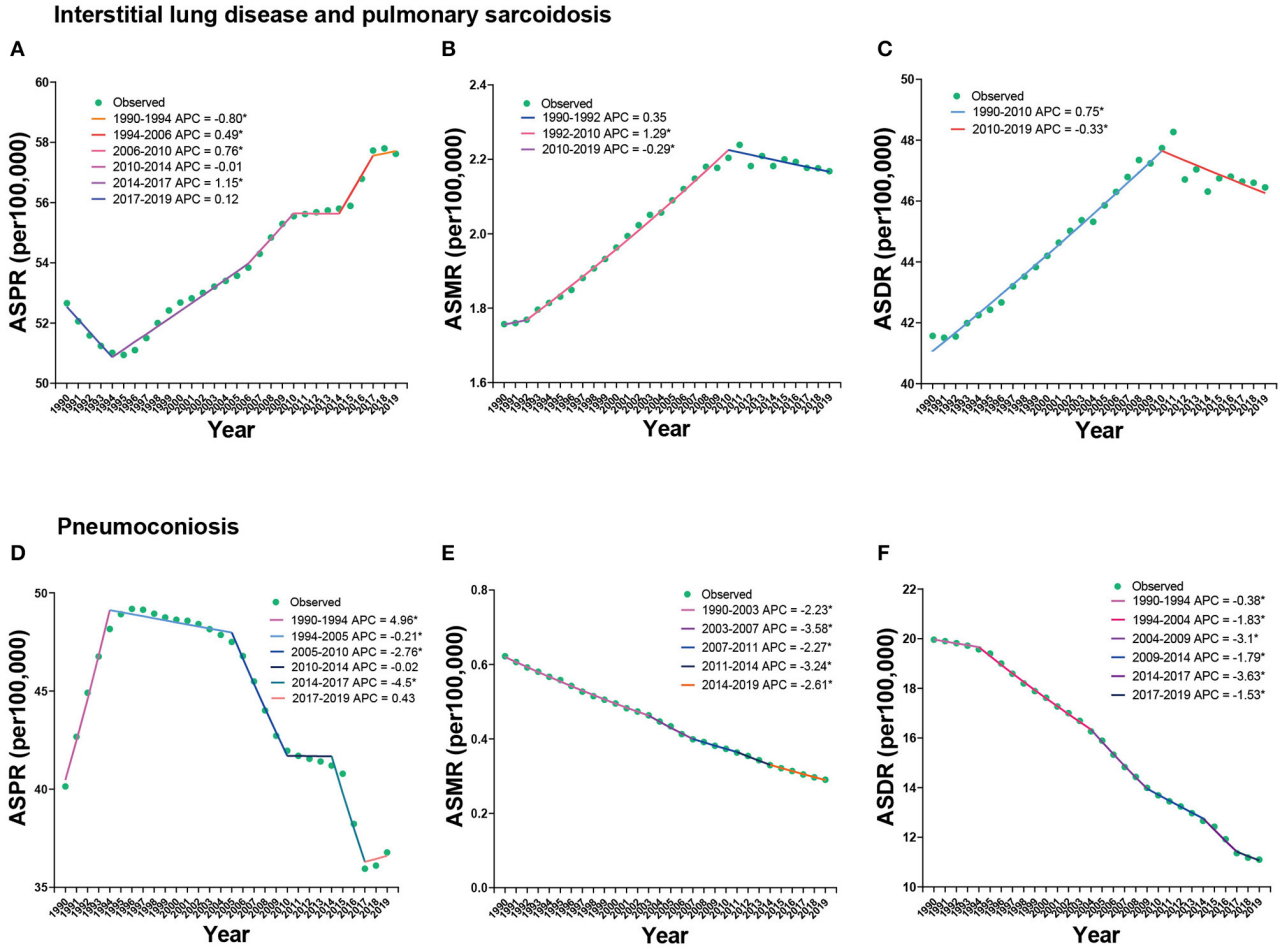
Joinpoint regression analysis of ASPR **(A, D)**, ASMR **(B, E)**, and ASDR **(C, F)** of ILDs from 1990 to 2019.

Figure 4 shows the Joinpoint regression analysis of the overall trends of ILDs. The ASPR of ILD increased from 1990 to 2019, with an AAPC of 0.32 (95% CI 0.19 to 0.46), but a mild descending trend was exhibited from 1990 to 1994, with the APC of −0.8 (95% CI −1.13 to −0.48) (Figure 4A). The ASMR of ILD increased from 1992 to 2010 and then decreased from 2010 to 2019, with APC of 1.29 (95% CI 1.23 to 1.34) and −0.29 (95% CI −0.45 to −0.14), respectively (Figure 4B). Similarly, the ASDR of ILD increased from 1990 to 2010 and then decreased until 2019 (Figure 4C). The ASPR of pneumoconiosis fluctuated, increasing from 1990 to 1994, with APC of 4.96 (95% CI 4.53 to 5.4), and then decreasing until 2017 (Figure 4D), whereas the ASMR and ASDR kept decreasing, with AAPC of −2.59 (95% CI −2.73 to −2.46) in ASMR and −2.01 (95% CI −2.12 to −1.9) in ASDR (Figures 4E, F). We further calculated the APCs and AAPCs of ILD in each sex, as shown in Supplementary Table 3. All AAPC values were between 0 and 1 and showed statistically significant differences. Nevertheless, the APCs of ASMR and ASDR in women from 2008 to 2019, which represented declining trends, were not statistically significant (Supplementary Table 3).

### 3.3. Descriptive analysis of the prevalence, mortality, and DALYs of ILD by age, period, and cohort

**Figure 5:**
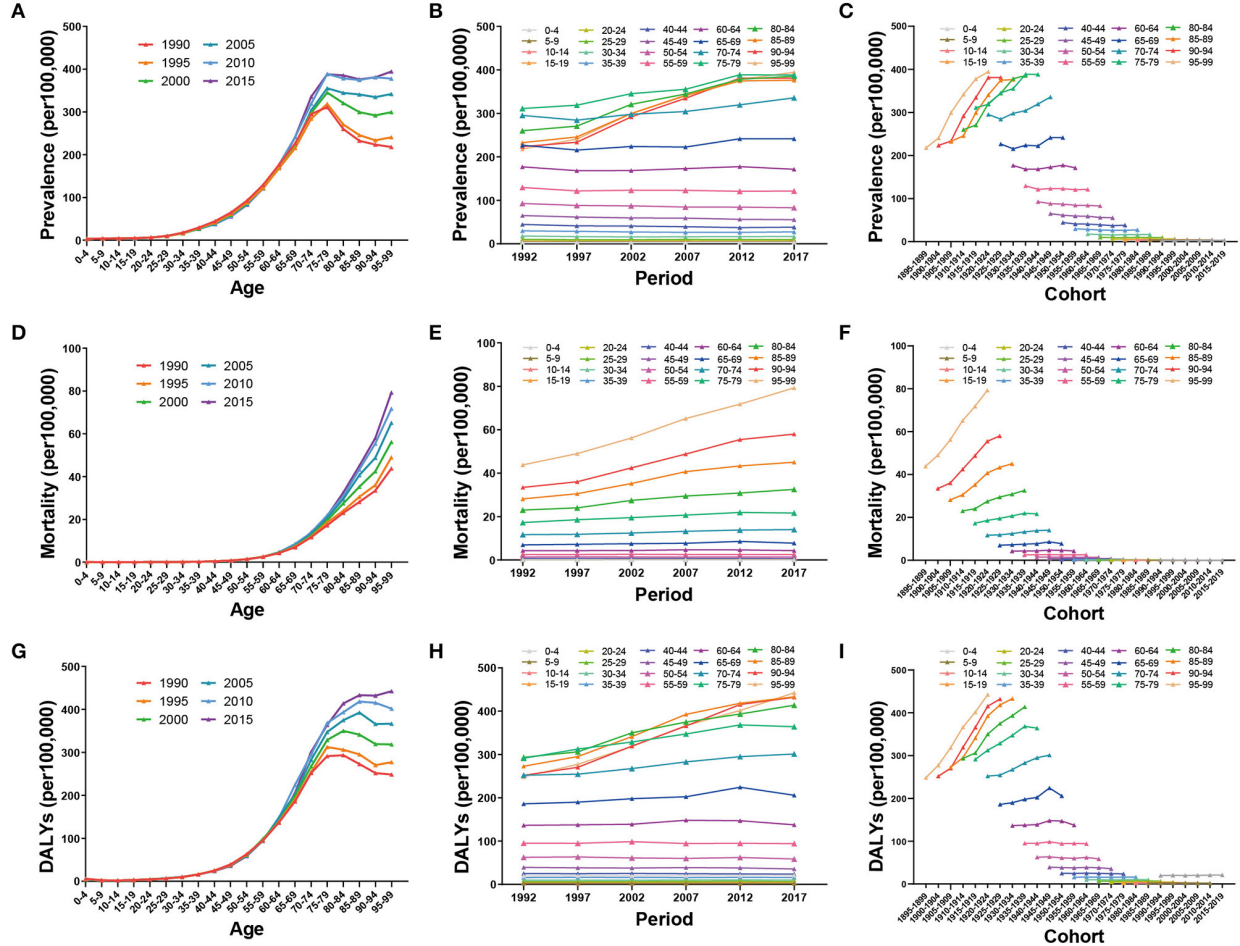
Global trends of prevalence, mortality, and DALYs by age **(A, D, G)**, period **(B, E, H)**, and cohort **(C, F, I)** of ILD from 1990 to 2019.

ASPR increased with increasing age in all periods, peaked in the age group of 75–79 years, and then slightly decreased or leveled off. In the age group of >70 years, ASPR increased over time, especially from 1997 to 2012, whereas in the other age groups, only subtle changes were noted. The most recent birth cohort had the lowest ASPR (Figures 5A–C). With increasing age, ASMR increased, and with time changes, it also increased in the older age groups. Similarly, the more recent birth cohort had a lower ASMR (Figures 5D–F). ASDR exhibited a similar trend with ASPR and ASMR, except for a slight increase in the most recent birth cohort (Figures 5G–I).

### 3.4. Age–period–cohort effects on the prevalence, mortality, and DALYs of ILD

**Figure 6:**
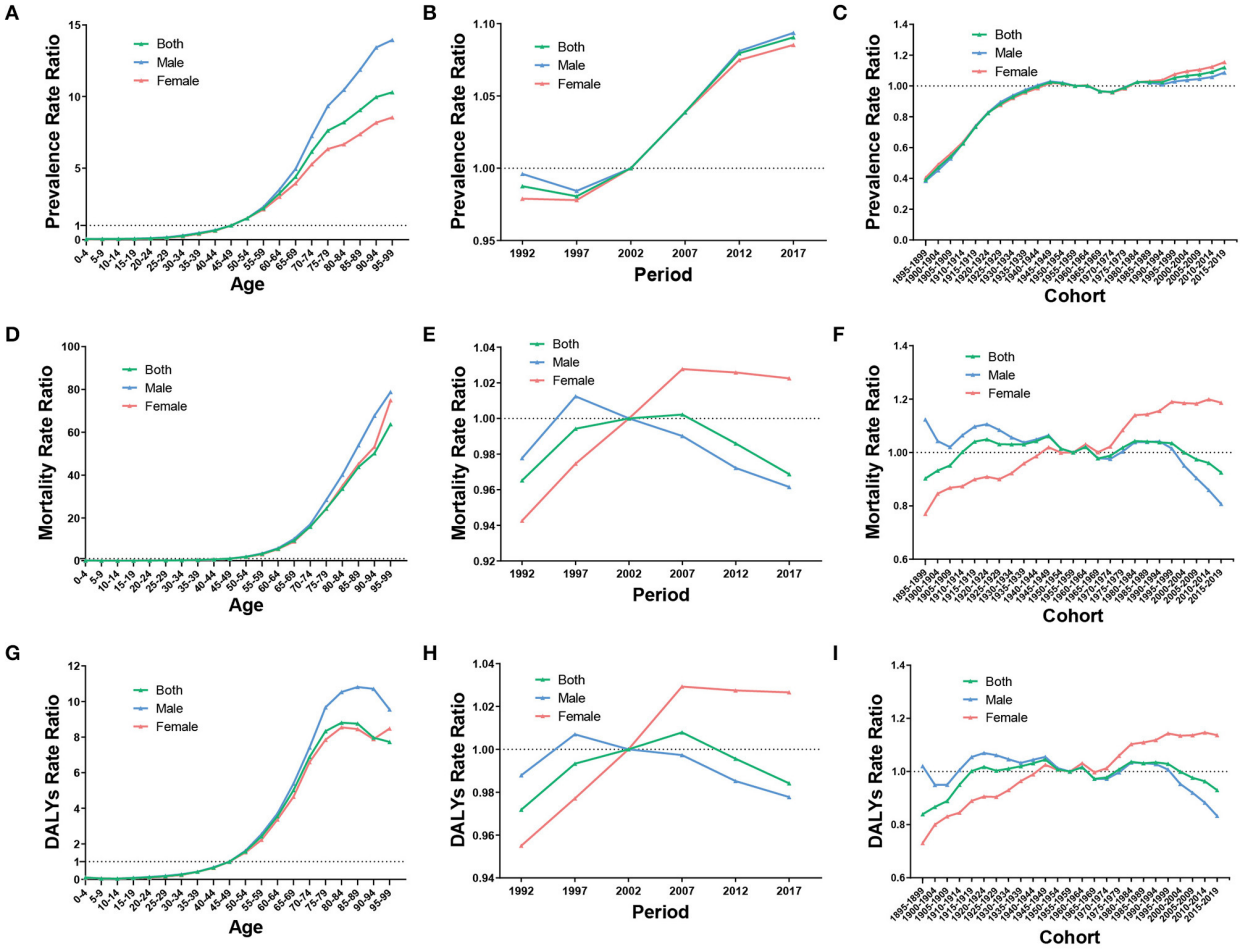
RR of prevalence, mortality, and DALYs due to age **(A, D, G)**, period **(B, E, H)**, and cohort **(C, F, I)** effect of ILD from 1990 to 2019.

After controlling for period and cohort effect, the RR of ASPR, ASMR, and ASDR increased rapidly with increasing age and particularly in the age group >55 years. The RR of ASPR, ASMR, and ASDR for both sexes in the 80–84 age group was 8.2 times (95% CI 8.06 to 8.34), 33.64 times (95% CI 33 to 34.29), and 8.81 times (95% CI 8.71 to 8.91) more than that of the reference group, respectively. Moreover, the RR of the age effect was higher in men than in women in most age groups (Figures 6A, D, G).

After controlling for age and cohort effect, the RR of ASPR increased over time, from 0.99 (95% CI 0.98 to 1) in 1992 to 1.09 (95% CI 1.08 to 1.1) in 2017, being relatively consistent in both sexes. In men, the RR of ASMR and ASDR increased from 1992 to 1997 and then decreased from 1997 to 2017, whereas in women, the RR increased from 1992 to 2007 and then slightly decreased from 2007 to 2017 (Figures 6B, E, H).

After controlling for age and period effect, the RR of ASPR exhibited an upward trend from the birth cohort 1895 to 1899 to the birth cohort 1950 to 1954, then the trend mildly fluctuated, and slightly increased until the most recent birth cohort. Regarding ASMR and ASDR, the RR presented a trend of fluctuating variation as the birth cohort changed. Intriguingly, men and women showed relatively opposite trends, from 1.12 (95% CI 0.94 to 1.34) to 0.81 (95% CI 0.63 to 1.04) in ASMR and from 1.02 (95% CI 0.82 to 1.28) to 0.83 (95% CI 0.77 to 0.9) in ASDR in men and from 0.77 (95% CI 0.67 to 0.89) to 1.19 (95% CI 0.91 to 1.55) in ASMR and from 0.73 (95% CI 0.64 to 0.84) to 1.14 (95% CI 1.06 to 1.21) in ASDR in women (Figures 6C, F, I).

The net and local drifts are provided in Supplementary Table 4. According to Wald's chi-square tests, the APC models generally showed significant differences (Supplementary Table 5).

### 3.5. Projections of the global trends of ILD until 2030

**Figure 7:**
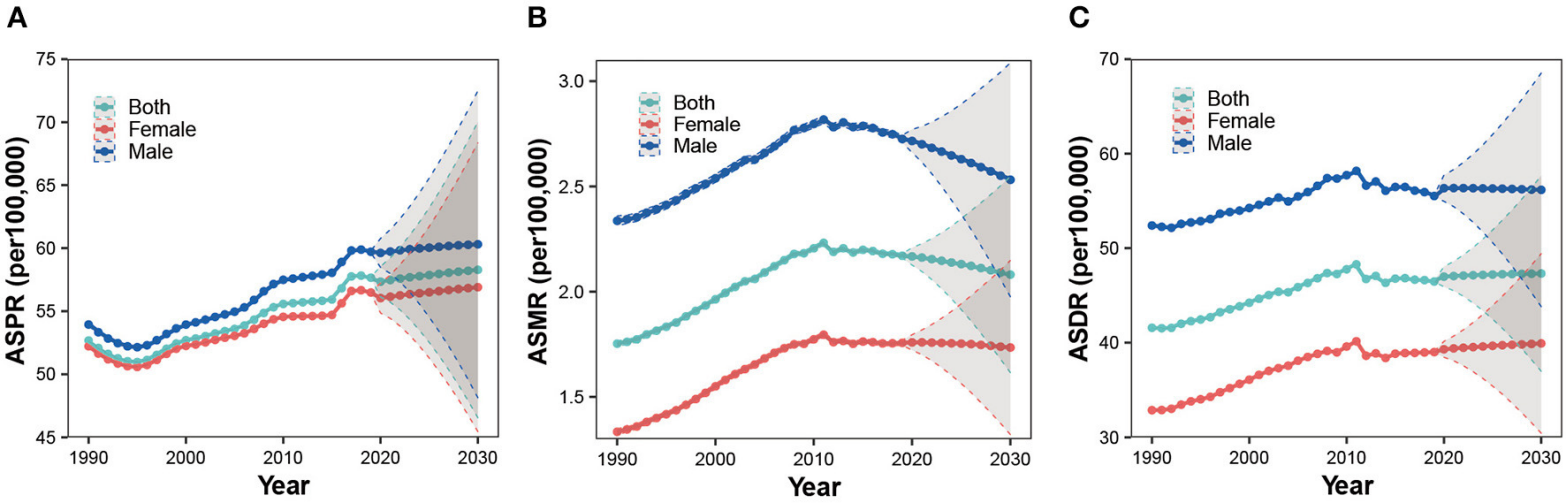
Projections of ASPR **(A)**, ASMR **(B)**, and ASDR **(C)** of ILD among both sexes, men and women, until 2030.

As presented in Figure 7, we projected that the ASPR, ASMR, and ASDR of ILD would stabilize with little variation over the next decade, with the rate higher in men than in women (Figures 7A–C). ASPR would slightly increase from 57.34 per 100,000 (95% CI 56.14 to 58.55) in 2020 to 58.28 per 100,000 (95% CI 46.54 to 70.02) in 2030; ASMR would mildly decrease from 2.17 per 100,000 (95% CI 2.13 to 2.21) in 2020 to 2.08 per 100,000 (95% CI 1.61 to 2.55) in 2030; and ASDR would slightly increase from 46.99 per 100,000 (95% CI 45.93 to 48.05) in 2020 to 47.32 per 100,000 (95% CI 36.97 to 57.66) in 2030.

## 4. Discussion

This study showed that the global ASPR, ASMR, and ASDR of ILD slightly increased or leveled off, whereas that of pneumoconiosis decreased from 1990 to 2019. Nevertheless, the prevalent cases, deaths, and DALYs numbers of ILD increased, whereas those of pneumoconiosis remained with little variation since first estimated in 1990, which is probably due to global population growth and population aging adjusted by age-standardized estimates (18). Despite global efforts made to alleviate the burden of chronic respiratory diseases, the burden of ILDs remains relatively severe and should not be neglected (24). However, the burden of pneumoconiosis has been mitigated over the last three decades, which is probably attributed to industrial injury compensation and many measures that have been implemented to protect workers from dust inhalation (25, 26). Nevertheless, the age-standardized incidence rate of asbestos has increased globally, despite the use of asbestos being completely banned in many countries, which indicates that asbestos regulation policies were not sufficient and effective (27).

For ILD, the high-SDI regions had the highest ASPR, whereas the low-middle and low-SDI regions had the highest ASMR and ASDR. Diagnostic tools such as computed tomography and multidisciplinary discussions are crucial for the diagnosis of ILD (28). Low income was correlated with a higher risk of comorbidities and adverse outcomes in patients with sarcoidosis (29). The availability and accessibility of abundant medical resources in high-SDI regions contributed to the diagnosis of ILD and perhaps led to the highest prevalence rate. Furthermore, a lack of appropriate diagnosis, optimal treatment, and adequate healthcare systems in low-middle and low-SDI regions may result in higher mortality and DALYs rates (30). Moreover, the black race is considered a risk factor for progressive fibrosis and death in systemic sclerosis-related interstitial lung disease and sarcoidosis (31). As the GARD proposed, low- and middle-income countries are especially in need to foster country-specific initiatives to alleviate the ILD burden (14). Notably, ASPR, ASMR, and ASDR increased the most in high-SDI regions despite robust healthcare services, which may indicate that either ILD was associated with many risk factors that have not been fully addressed or the present provisioned resources are still insufficient (18).

Regarding pneumoconiosis, the middle-SDI regions had the highest ASPR, ASMR, and ASDR, followed by the high-middle SDI regions. Occupational exposure to asbestos, silica, and coal dust is closely associated with pneumoconiosis. Many substitutes have replaced asbestos, and measures have been taken to control asbestos imports in many developed countries. Russia, China, and Kazakhstan rank among the top three asbestos producers, possibly due to the low cost and tensile strength of asbestos (32). Moreover, silicosis is a serious problem, particularly in developing countries, and is often underreported due to inadequate surveillance (33). Finding suitable alternatives, implementing preventive policies, and improving the workplace environment are of great significance in these regions.

In 2019, Bolivia had the highest ASMR for ILD, followed by Peru; ASDR in Bolivia and Peru also ranked ahead among the 204 countries and territories. Furthermore, ASPR, ASMR, and ASDR in the two countries showed an upward trend from 1990 to 2019. Both located in South America, Bolivia, and Peru are well-known for their abundant mineral resources, particularly Bolivia. Although natural resources are advantageous in these two developing countries, public health is alarming. Workplace exposure contributes substantially to the burden of multiple chronic respiratory diseases, such as IPF, for instance, has a population-attributable fraction of 26% (34). In addition to the high ILD burden, participants in a study living in proximity to mining sites near Potosi, Bolivia, had higher frequencies of hypertension, hematuria, and ketonuria, and the majority of adobe brick houses in Potosi, Bolivia, contained concentrations of bio-accessible Pb and As, which represent a potential health risk (35, 36). Therefore, effective measures are urgently required to protect residents from contaminated environments.

The global prevalence, mortality, and DALYs of ILDs were higher in men than in women, which was consistent with the findings of previous studies (8, 37). However, CTD-ILD was more prevalent in women, possibly because they are more susceptible to connective tissue disease (38). Tobacco smoking has an independent detrimental effect on IPF, and alveolar wall fibrosis occurs in smokers; historically, men smoke more than women, which may account for the higher burden of ILDs in men (39, 40). Sex hormone regulation also plays a vital role in pro-inflammatory and pro-fibrotic factors, with estrogens enhancing inflammation and remodeling, whereas androgens may have the opposite effect (41). Moreover, most workers exposed to toxic particles in the workplace were men, the sociologically dominant labor force.

Age effects showed a high RR of prevalence, mortality, and DALYs in older adults. Some ILDs occur secondary to drugs, therapies, and connective tissue diseases, and the prevalence of connective tissue diseases remains high in older adults due to longer life expectancies and better tolerated treatments, which may lead to a high RR of prevalence in the older age groups (42). Moreover, age-related telomere shortening, protein folding, and oxidation may damage the alveolar epithelium in IPF, which is strongly correlated with older age (43). Comorbidities play a significant role in the dramatic increase in the RR of mortality and DALYs in older adults. Chronic obstructive pulmonary disease, cardiovascular diseases, and autoimmune disorders are common in older adults with ILD, and chronic respiratory diseases are highly associated with aging (44–46). Furthermore, relatively bad medical adherence and depression in older adults may also affect the prognosis of ILD (47, 48). Age effects have impacted the slightly elevated trends of ILD over the last three decades, associated with global demographic changes in which the proportion of the population over 70 years increased from 3.77% in 1990 to 5.99% in 2019 (49). An increase in the proportion of the population with high RR results in an elevated trend of ILD (50). Chronic disabling illness secondary to ILD in older adults might be preventable through patient-centered care aiming to improve the quality of life and decrease the use of health services (51).

Period effects showed that the RR of prevalence increased over time and was above the average risk after 2002. In terms of mortality and DALYs, the RR increased before 2007 and then decreased for the total population. From the perspective of global social development, economic growth stimulated medical advances such as imaging and functional tests; thus, the diagnosis of ILD may be more precise and common, as many ILDs are rare and difficult to diagnose, resulting in an increased RR of prevalence. Global achievements in tobacco smoking may explain the decreased RR of mortality and DALYs in men (52). Notably, the GARD was launched in 2007, and more than 20 countries have initiated activities since then, which may have contributed to the decreased RR in the total population (53). Furthermore, reduction in air pollution, prevention of allergen contact, improvement in the workplace environment, and enhancement of public health awareness may also affect the trends of ILD (20).

Cohort effects showed that the RR of prevalence increased with the birth cohort more recently, whereas that of mortality and DALYs fluctuated strikingly, with men showing decreased RR, women, increased RR, and the total population, leveled off RR. Smoking cessation in men and a higher prevalence of connective tissue disease in women may be reasons for this sex disparity. Despite the ILD burden being heavier in men, the RR in women had increased as the birth cohort changed, and women were also supposed to pay adequate attention to ILD. Genetic background and epigenetic modifications have been identified as important factors in fibrotic lung diseases, among which the MUC5B promoter polymorphism is a common gene variant (54). It is difficult to explain the variation in the cohort effect. Perhaps genetics, exposure to a wide range of environmental risk factors, eating and living lifestyles, and mental health all contribute to the cohort effects of ILD.

We projected that the ASPR, ASMR, and ASDR of ILD would stabilize with mild variations over the next decade. Given that population aging is a global phenomenon, an increase in the proportion of older adults would result in an increasing prevalence. Medical advances, such as imaging, may also lead to an upward trend in ILD prevalence. As for mortality, in addition to medical developments, global achievements in tobacco smoking would also account for a downward trend, especially in men. As mentioned above, ILD remains a global health challenge, and the alleviation of the ILD burden is of great significance. Patient education, decision-sharing, smoking cessation, and pneumococcal and influenza vaccines are essential for ILD management (31). Treatments aimed at ameliorating the disease or retarding its progression while improving or maintaining the quality of life are recommended, including pulmonary rehabilitation (55).

Compared with previous studies on ILDs epidemiology that mainly focused on a specific form of ILDs and localized in a certain region, our study used data retrieved from the GBD database, providing a global perspective to make comparisons between different countries and territories. As many ILDs are associated with occupational exposure, our study may offer some guidance for policymakers in implementing control regulations, especially in low- and middle-SDI regions. Moreover, this is the first study to analyze the independent effects of age, period, and cohort on ILD prevalence, mortality, and DALYs and found that the age effect played a crucial role in ILD. For countries confronting population aging, the social burden of chronic diseases such as ILD should be seriously considered.

This study had some limitations. First, because ILDs encompass a variety of diseases, their classification and diagnosis remain difficult, and the epidemiology may vary significantly in each kind of ILDs. Thus, more studies on the epidemiology of a specific kind of ILDs are expected. Second, our prediction model based on previous data may not have been founded on current political realities; for instance, the undercutting of regulations on inhalation exposure may result in an increasing trend of ILDs. Third, the input prevalence data for the GBD study were primarily derived from hospital inpatient records and insurance claims; however, claims data can be unreliable (18). In counties with low- or low-middle SDI, a lack of medical expertise and equipment may result in undiagnosed and unregistered cases. Finally, as some counties are vast, the evaluation of the disease burden at the country and territory levels may have varied considerably between different provinces. Therefore, large cohort studies are needed in each region to measure the local burden of ILDs.

## 5. Conclusion

The ASPR, ASMR, and ASDR slightly increased for ILDs and decreased for pneumoconiosis from 1990 to 2019. In ILDs, the high-SDI regions possessed the highest ASPR, whereas the low-middle SDI regions had the highest ASMR and ASDR, followed by the low-SDI regions, possibly because of the disequilibrium of medical resources. For pneumoconiosis, the middle-SDI region had the highest ASPR, ASMR, and ASDR. Control regulations for reducing occupational exposures are needed in these regions. Older adults have a high risk of ILD, and the age effect played a significant role in the increase of ASPR, considering the global population aging. The global trends of ILDs would stabilize with minimum variation and remain relatively high over the next decade. Global actions and country-specific initiatives are needed to mitigate the burden of ILDs.

## Data availability statement

The original contributions presented in the study are publicly available. This data can be found here: https://vizhub.healthdata.org/gbd-results/.

## Author contributions

DJ and QZ conceived the study. QZ analyzed the data and wrote the manuscript. All authors approved the submitted version.

## Supplementary material

Supplementary Figure 1 EAPC for prevalence **(A)**, mortality **(B)**, and DALYs **(C)** of pneumoconiosis in 204 countries and territories from 1990 to 2019.

Supplementary Table 1 ASPR, ASMR, and ASDR (per 100,000) of ILD, and SDIs in 204 countries and territories in 1990.

Supplementary Table 2 ASPR, ASMR, and ASDR (per 100,000) of ILD, and SDIs in 204 countries and territories in 2019.

Supplementary Table 3 Joinpoint regression analysis for global trends of ASPR, ASMR, and ASDR (per 100,000) of ILD among both sexes, males, and females from 1990 to 2019.

Supplementary Table 4 Local drifts and net drifts for global trends of ASPR, ASMR, and ASDR (per 100,000) of ILD among both sexes, males, and females from 1990 to 2019.

Supplementary Table 5 Wald Chi-Square tests for estimable functions in the APC model.
