# Supplementary figures and images for: Global trends of interstitial lung diseases from 1990 to 2019: an age–period–cohort study based on the Global Burden of Disease study 2019, and projections until 2030
Source: Front Med (Lausanne). 2023 Jul 24;10:1141372. doi: 10.3389/fmed.2023.1141372 (PMC10404716; doi:10.3389/fmed.2023.1141372)

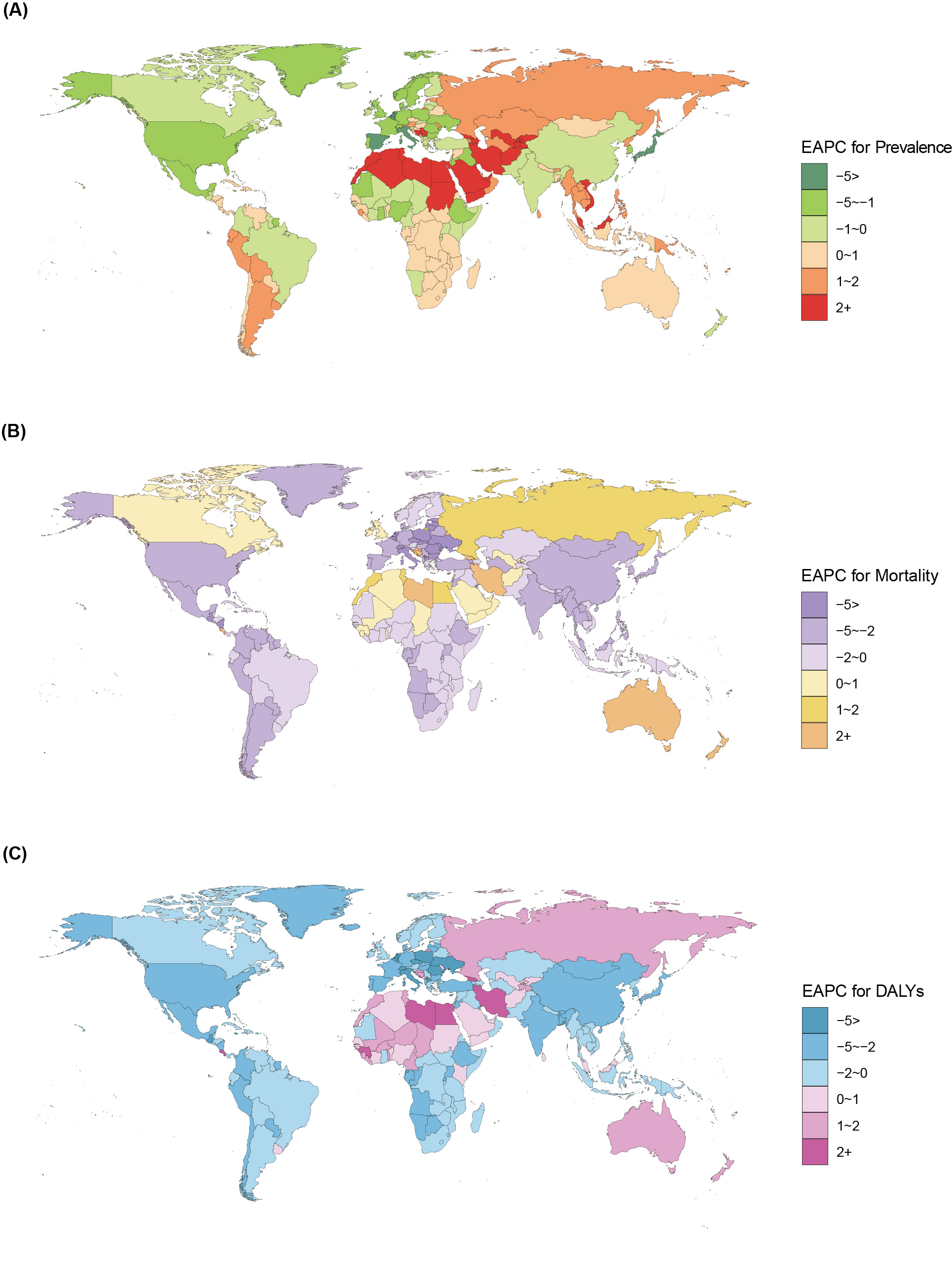

Supplement: Supplementary Figure 1 — EAPC for prevalence (A), mortality (B), and DALYs (C) of pneumoconiosis in 204 countries and territories from 1990 to 2019. [file Image_1.JPEG]
