# Supplementary material for: Global trends of interstitial lung diseases from 1990 to 2019: an age–period–cohort study based on the Global Burden of Disease study 2019, and projections until 2030
Source: Front Med (Lausanne). 2023 Jul 24;10:1141372. doi: 10.3389/fmed.2023.1141372 (PMC10404716; doi:10.3389/fmed.2023.1141372)
Supplement: Supplementary Table 1 — ASPR, ASMR, and ASDR (per 100,000) of ILD, and SDIs in 204 countries and territories in 1990. [file Table_1.DOCX]

Supplementary Table S1: ASPR, ASMR, and ASDR (per 100,000) of ILD, and SDIs in 204 countries and territories in 1990

| Location | ASPR | ASMR | ASDR | SDI | SDI level |
| --- | --- | --- | --- | --- | --- |
| Afghanistan | 26.53 | 1.16 | 26.2 | 0.187 | Low SDI |
| Albania | 47.44 | 1.48 | 33.78 | 0.54 | Low-middle SDI |
| Algeria | 28.98 | 0.98 | 21.52 | 0.436 | Low SDI |
| American Samoa | 43.41 | 1.33 | 50.64 | 0.606 | Low-middle SDI |
| Andorra | 43.39 | 2.09 | 44.61 | 0.834 | High SDI |
| Angola | 22.59 | 1.36 | 30.67 | 0.238 | Low SDI |
| Antigua and Barbuda | 16.98 | 0.42 | 10.1 | 0.579 | Low-middle SDI |
| Argentina | 53.64 | 2.62 | 58.34 | 0.581 | Low-middle SDI |
| Armenia | 55.28 | 2.17 | 49.9 | 0.536 | Low-middle SDI |
| Australia | 41.57 | 1.22 | 26.47 | 0.738 | High-middle SDI |
| Austria | 53.94 | 0.70 | 20.03 | 0.753 | High-middle SDI |
| Azerbaijan | 29.00 | 1.76 | 39.68 | 0.576 | Low-middle SDI |
| Bahrain | 41.24 | 2.23 | 44.39 | 0.553 | Low-middle SDI |
| Bangladesh | 47.54 | 3.98 | 91.56 | 0.267 | Low SDI |
| Barbados | 22.37 | 0.92 | 21.2 | 0.649 | Middle SDI |
| Belarus | 46.37 | 0.83 | 23.36 | 0.591 | Low-middle SDI |
| Belgium | 35.82 | 1.12 | 25.47 | 0.746 | High-middle SDI |
| Belize | 42.76 | 2.54 | 65.73 | 0.428 | Low SDI |
| Benin | 21.90 | 1.16 | 26.58 | 0.209 | Low SDI |
| Bermuda | 47.16 | 3.31 | 73.57 | 0.685 | Middle SDI |
| Bhutan | 40.70 | 3.51 | 75.31 | 0.228 | Low SDI |
| Bolivia | 64.60 | 7.76 | 146.76 | 0.412 | Low SDI |
| Bosnia and Herzegovina | 43.66 | 1.05 | 25.95 | 0.533 | Low-middle SDI |
| Botswana | 42.00 | 2.76 | 58.45 | 0.431 | Low SDI |
| Brazil | 31.49 | 1.03 | 26.69 | 0.487 | Low-middle SDI |
| Brunei | 159.96 | 4.82 | 113.21 | 0.676 | Middle SDI |
| Bulgaria | 40.94 | 0.53 | 16.72 | 0.631 | Middle SDI |
| Burkina Faso | 18.42 | 0.65 | 15.72 | 0.125 | Low SDI |
| Burundi | 21.25 | 1.36 | 32.57 | 0.198 | Low SDI |
| Cambodia | 9.90 | 0.53 | 11.83 | 0.266 | Low SDI |
| Cameroon | 25.77 | 1.45 | 32.91 | 0.313 | Low SDI |
| Canada | 88.05 | 2.06 | 49.23 | 0.79 | High-middle SDI |
| Cape Verde | 19.84 | 0.62 | 16.45 | 0.292 | Low SDI |
| Central African Republic | 23.91 | 2.01 | 44.13 | 0.186 | Low SDI |
| Chad | 21.38 | 1.25 | 28.63 | 0.108 | Low SDI |
| Chile | 82.09 | 4.34 | 89.23 | 0.592 | Low-middle SDI |
| China | 30.06 | 0.47 | 13.22 | 0.433 | Low SDI |
| Colombia | 26.08 | 0.43 | 12.08 | 0.478 | Low-middle SDI |
| Comoros | 21.25 | 1.20 | 27.13 | 0.274 | Low SDI |
| Congo (Brazzaville) | 25.24 | 1.95 | 42.12 | 0.364 | Low SDI |
| Cook Islands | 47.42 | 1.71 | 66.37 | 0.625 | Middle SDI |
| Costa Rica | 53.41 | 2.22 | 54.67 | 0.532 | Low-middle SDI |
| Cote d'Ivoire | 21.52 | 1.18 | 26.67 | 0.256 | Low SDI |
| Croatia | 47.45 | 0.31 | 12.52 | 0.68 | Middle SDI |
| Cuba | 16.34 | 0.27 | 9.46 | 0.578 | Low-middle SDI |
| Cyprus | 56.36 | 3.90 | 71.45 | 0.662 | Middle SDI |
| Czech Republic | 37.67 | 0.53 | 16.75 | 0.688 | Middle SDI |
| Denmark | 38.90 | 1.43 | 35.79 | 0.806 | High SDI |
| Djibouti | 22.51 | 1.10 | 26.12 | 0.275 | Low SDI |
| Dominica | 19.74 | 0.86 | 20.42 | 0.579 | Low-middle SDI |
| Dominican Republic | 14.58 | 0.37 | 11.05 | 0.425 | Low SDI |
| DR Congo | 25.17 | 1.76 | 37.82 | 0.26 | Low SDI |
| Ecuador | 39.36 | 3.50 | 67.33 | 0.503 | Low-middle SDI |
| Egypt | 29.74 | 1.12 | 27.36 | 0.403 | Low SDI |
| El Salvador | 29.79 | 1.28 | 33.81 | 0.39 | Low SDI |
| Equatorial Guinea | 20.32 | 1.71 | 37.54 | 0.208 | Low SDI |
| Eritrea | 20.09 | 1.15 | 28.38 | 0.198 | Low SDI |
| Estonia | 45.01 | 0.65 | 23.67 | 0.665 | Middle SDI |
| eSwatini | 39.68 | 2.62 | 56.27 | 0.392 | Low SDI |
| Ethiopia | 17.53 | 0.98 | 23.71 | 0.144 | Low SDI |
| Federated States of Micronesia | 51.91 | 2.82 | 104.76 | 0.447 | Low SDI |
| Fiji | 35.88 | 0.76 | 29.12 | 0.527 | Low-middle SDI |
| Finland | 44.95 | 1.44 | 32.56 | 0.757 | High-middle SDI |
| France | 35.68 | 1.09 | 24.99 | 0.738 | High-middle SDI |
| Gabon | 26.15 | 1.58 | 34.78 | 0.388 | Low SDI |
| Georgia | 30.33 | 0.99 | 24.69 | 0.654 | Middle SDI |
| Germany | 37.98 | 1.37 | 32.44 | 0.819 | High SDI |
| Ghana | 20.18 | 1.59 | 34.82 | 0.355 | Low SDI |
| Greece | 23.21 | 0.48 | 12.28 | 0.682 | Middle SDI |
| Greenland | 110.98 | 4.83 | 107.58 | 0.655 | Middle SDI |
| Grenada | 20.39 | 0.93 | 23.11 | 0.463 | Low-middle SDI |
| Guam | 113.03 | 4.79 | 146.11 | 0.693 | High-middle SDI |
| Guatemala | 36.63 | 2.31 | 56.14 | 0.315 | Low SDI |
| Guinea | 20.83 | 1.04 | 23.94 | 0.175 | Low SDI |
| Guinea-Bissau | 21.12 | 1.31 | 30.53 | 0.2 | Low SDI |
| Guyana | 19.21 | 1.11 | 26.74 | 0.452 | Low SDI |
| Haiti | 17.43 | 2.05 | 46.64 | 0.307 | Low SDI |
| Honduras | 37.26 | 2.31 | 60.29 | 0.33 | Low SDI |
| Hungary | 46.37 | 0.95 | 27.72 | 0.659 | Middle SDI |
| Iceland | 38.69 | 1.00 | 23.41 | 0.764 | High-middle SDI |
| India | 77.23 | 5.79 | 119.65 | 0.327 | Low SDI |
| Indonesia | 14.21 | 0.78 | 17.71 | 0.452 | Low SDI |
| Iran | 27.75 | 0.54 | 13.15 | 0.404 | Low SDI |
| Iraq | 27.87 | 0.59 | 15.7 | 0.392 | Low SDI |
| Ireland | 50.10 | 2.09 | 45.56 | 0.73 | High-middle SDI |
| Israel | 30.73 | 1.28 | 28.77 | 0.717 | High-middle SDI |
| Italy | 53.65 | 0.52 | 16.28 | 0.712 | High-middle SDI |
| Jamaica | 17.19 | 0.45 | 13.5 | 0.542 | Low-middle SDI |
| Japan | 153.45 | 2.27 | 58.24 | 0.791 | High-middle SDI |
| Jordan | 58.86 | 3.51 | 73.06 | 0.52 | Low-middle SDI |
| Kazakhstan | 26.60 | 0.78 | 22.82 | 0.602 | Low-middle SDI |
| Kenya | 25.06 | 1.00 | 23.65 | 0.333 | Low SDI |
| Kiribati | 40.52 | 1.52 | 57.15 | 0.425 | Low SDI |
| Kuwait | 47.53 | 1.92 | 38.84 | 0.655 | Middle SDI |
| Kyrgyzstan | 25.81 | 0.54 | 14.71 | 0.532 | Low-middle SDI |
| Laos | 11.00 | 0.91 | 20.78 | 0.268 | Low SDI |
| Latvia | 60.49 | 1.07 | 33.73 | 0.675 | Middle SDI |
| Lebanon | 30.48 | 0.92 | 20.95 | 0.462 | Low-middle SDI |
| Lesotho | 36.51 | 2.34 | 49.93 | 0.321 | Low SDI |
| Liberia | 18.98 | 0.59 | 14.64 | 0.221 | Low SDI |
| Libya | 32.20 | 0.96 | 21.97 | 0.405 | Low SDI |
| Lithuania | 35.05 | 0.40 | 14.38 | 0.67 | Middle SDI |
| Luxembourg | 32.28 | 0.94 | 21.7 | 0.815 | High SDI |
| Madagascar | 24.02 | 1.44 | 35.14 | 0.265 | Low SDI |
| Malawi | 19.62 | 0.91 | 21.77 | 0.213 | Low SDI |
| Malaysia | 16.48 | 1.01 | 22.56 | 0.542 | Low-middle SDI |
| Maldives | 89.64 | 9.67 | 213.25 | 0.303 | Low SDI |
| Mali | 22.46 | 1.18 | 29.63 | 0.126 | Low SDI |
| Malta | 51.38 | 2.14 | 45.48 | 0.666 | Middle SDI |
| Marshall Islands | 54.22 | 2.49 | 90.82 | 0.398 | Low SDI |
| Mauritania | 22.41 | 1.07 | 24.99 | 0.308 | Low SDI |
| Mauritius | 44.65 | 3.36 | 67.76 | 0.527 | Low-middle SDI |
| Mexico | 57.60 | 1.91 | 46.43 | 0.507 | Low-middle SDI |
| Moldova | 38.73 | 0.28 | 11.45 | 0.585 | Low-middle SDI |
| Monaco | 46.79 | 1.61 | 35.7 | 0.834 | High SDI |
| Mongolia | 33.89 | 2.17 | 73.47 | 0.465 | Low-middle SDI |
| Montenegro | 40.84 | 0.27 | 10.37 | 0.701 | High-middle SDI |
| Morocco | 26.65 | 0.83 | 18.49 | 0.347 | Low SDI |
| Mozambique | 18.11 | 0.72 | 17.04 | 0.12 | Low SDI |
| Myanmar | 16.47 | 1.40 | 30.41 | 0.284 | Low SDI |
| Namibia | 39.15 | 2.83 | 58.78 | 0.454 | Low SDI |
| Nauru | 51.38 | 2.92 | 117.45 | 0.499 | Low-middle SDI |
| Nepal | 47.67 | 7.44 | 153.55 | 0.198 | Low SDI |
| Netherlands | 31.63 | 0.65 | 16.2 | 0.796 | High-middle SDI |
| New Zealand | 51.78 | 1.38 | 30.39 | 0.757 | High-middle SDI |
| Nicaragua | 25.28 | 0.66 | 17.03 | 0.338 | Low SDI |
| Niger | 23.09 | 1.47 | 33.7 | 0.073 | Low SDI |
| Nigeria | 24.25 | 0.95 | 22.18 | 0.305 | Low SDI |
| Niue | 48.03 | 2.23 | 90.17 | 0.566 | Low-middle SDI |
| North Korea | 24.46 | 0.61 | 16.32 | 0.431 | Low SDI |
| North Macedonia | 38.60 | 0.36 | 12.62 | 0.618 | Middle SDI |
| Northern Mariana Islands | 108.56 | 3.71 | 127.1 | 0.692 | High-middle SDI |
| Norway | 81.37 | 1.30 | 33.79 | 0.807 | High SDI |
| Oman | 28.43 | 1.41 | 32.84 | 0.441 | Low SDI |
| Pakistan | 37.81 | 3.66 | 77.41 | 0.247 | Low SDI |
| Palau | 106.74 | 4.71 | 161 | 0.621 | Middle SDI |
| Palestine | 80.98 | 5.06 | 110.77 | 0.314 | Low SDI |
| Panama | 32.43 | 1.11 | 29.48 | 0.544 | Low-middle SDI |
| Papua New Guinea | 74.57 | 2.98 | 103.47 | 0.292 | Low SDI |
| Paraguay | 14.63 | 0.69 | 16.52 | 0.465 | Low-middle SDI |
| Peru | 89.18 | 8.94 | 174.87 | 0.501 | Low-middle SDI |
| Philippines | 10.52 | 0.36 | 8.36 | 0.497 | Low-middle SDI |
| Poland | 56.79 | 0.80 | 25.33 | 0.632 | Middle SDI |
| Portugal | 29.29 | 1.02 | 24.02 | 0.607 | Low-middle SDI |
| Puerto Rico | 30.76 | 1.40 | 33.39 | 0.67 | Middle SDI |
| Qatar | 29.84 | 0.87 | 19.86 | 0.585 | Low-middle SDI |
| Romania | 77.59 | 2.99 | 75.54 | 0.625 | Middle SDI |
| Russia | 39.55 | 0.46 | 15.09 | 0.695 | High-middle SDI |
| Rwanda | 21.07 | 1.37 | 32.75 | 0.257 | Low SDI |
| Saint Kitts and Nevis | 24.74 | 1.38 | 31.56 | 0.583 | Low-middle SDI |
| Saint Lucia | 24.86 | 1.43 | 33.34 | 0.483 | Low-middle SDI |
| Saint Vincent and the Grenadines | 16.74 | 0.47 | 12.11 | 0.462 | Low-middle SDI |
| Samoa | 53.29 | 2.61 | 98.84 | 0.531 | Low-middle SDI |
| San Marino | 26.14 | 0.40 | 10.39 | 0.814 | High SDI |
| Sao Tome and Principe | 28.24 | 2.00 | 44.44 | 0.299 | Low SDI |
| Saudi Arabia | 69.38 | 5.62 | 102.85 | 0.48 | Low-middle SDI |
| Senegal | 21.34 | 1.21 | 27.17 | 0.227 | Low SDI |
| Serbia | 33.41 | 0.52 | 16.18 | 0.626 | Middle SDI |
| Seychelles | 12.50 | 0.62 | 14.09 | 0.567 | Low-middle SDI |
| Sierra Leone | 20.03 | 1.00 | 22.68 | 0.207 | Low SDI |
| Singapore | 94.82 | 0.71 | 28.98 | 0.688 | Middle SDI |
| Slovakia | 41.37 | 0.56 | 18.94 | 0.656 | Middle SDI |
| Slovenia | 48.09 | 0.50 | 16.05 | 0.726 | High-middle SDI |
| Solomon Islands | 41.14 | 2.06 | 67.87 | 0.279 | Low SDI |
| Somalia | 21.07 | 1.35 | 31.85 | 0.051 | Low SDI |
| South Africa | 62.79 | 2.73 | 60.08 | 0.552 | Low-middle SDI |
| South Korea | 52.88 | 1.11 | 28.95 | 0.686 | Middle SDI |
| South Sudan | 22.60 | 1.33 | 31.02 | 0.248 | Low SDI |
| Spain | 52.43 | 2.03 | 44.97 | 0.647 | Middle SDI |
| Sri Lanka | 13.71 | 0.74 | 18.42 | 0.504 | Low-middle SDI |
| Sudan | 27.21 | 1.19 | 27.09 | 0.227 | Low SDI |
| Suriname | 20.78 | 1.14 | 27.36 | 0.498 | Low-middle SDI |
| Sweden | 55.26 | 1.36 | 31.54 | 0.769 | High-middle SDI |
| Switzerland | 40.69 | 1.37 | 31.39 | 0.868 | High SDI |
| Syria | 33.49 | 1.26 | 27.22 | 0.367 | Low SDI |
| Taiwan (province of China) | 15.38 | 0.37 | 9.44 | 0.667 | Middle SDI |
| Tajikistan | 88.90 | 5.68 | 115 | 0.468 | Low-middle SDI |
| Tanzania | 19.71 | 0.85 | 20.6 | 0.26 | Low SDI |
| Thailand | 11.48 | 0.42 | 10.67 | 0.508 | Low-middle SDI |
| The Bahamas | 28.43 | 1.48 | 35.01 | 0.692 | High-middle SDI |
| The Gambia | 21.84 | 1.09 | 24.86 | 0.218 | Low SDI |
| Timor-Leste | 11.02 | 0.82 | 18.4 | 0.274 | Low SDI |
| Togo | 22.20 | 1.10 | 25.39 | 0.266 | Low SDI |
| Tokelau | 39.81 | 2.26 | 85.27 | 0.427 | Low SDI |
| Tonga | 44.32 | 1.47 | 54.88 | 0.51 | Low-middle SDI |
| Trinidad and Tobago | 26.23 | 1.54 | 38.72 | 0.618 | Middle SDI |
| Tunisia | 29.39 | 0.84 | 19.64 | 0.434 | Low SDI |
| Turkey | 33.04 | 1.21 | 32.01 | 0.473 | Low-middle SDI |
| Turkmenistan | 27.67 | 1.25 | 30.94 | 0.548 | Low-middle SDI |
| Tuvalu | 39.86 | 2.86 | 113.83 | 0.426 | Low SDI |
| Uganda | 21.71 | 1.52 | 34.32 | 0.167 | Low SDI |
| UK | 67.80 | 2.05 | 46.59 | 0.745 | High-middle SDI |
| Ukraine | 67.38 | 1.39 | 40.34 | 0.653 | Middle SDI |
| United Arab Emirates | 35.58 | 1.57 | 40.08 | 0.621 | Middle SDI |
| Uruguay | 44.30 | 1.54 | 34.79 | 0.581 | Low-middle SDI |
| USA | 117.30 | 2.54 | 65.04 | 0.768 | High-middle SDI |
| Uzbekistan | 68.18 | 2.98 | 69.17 | 0.49 | Low-middle SDI |
| Vanuatu | 46.12 | 2.49 | 89.17 | 0.361 | Low SDI |
| Venezuela | 29.92 | 0.95 | 23.39 | 0.509 | Low-middle SDI |
| Vietnam | 11.98 | 0.83 | 17.33 | 0.39 | Low SDI |
| Virgin Islands | 29.70 | 1.95 | 44.5 | 0.667 | Middle SDI |
| Yemen | 27.30 | 1.06 | 24.02 | 0.176 | Low SDI |
| Zambia | 20.80 | 1.13 | 26.34 | 0.299 | Low SDI |
| Zimbabwe | 32.67 | 0.77 | 19.16 | 0.394 | Low SDI |
