# Supplementary material for: Global trends of interstitial lung diseases from 1990 to 2019: an age–period–cohort study based on the Global Burden of Disease study 2019, and projections until 2030
Source: Front Med (Lausanne). 2023 Jul 24;10:1141372. doi: 10.3389/fmed.2023.1141372 (PMC10404716; doi:10.3389/fmed.2023.1141372)
Supplement: Supplementary Table 2 — ASPR, ASMR, and ASDR (per 100,000) of ILD, and SDIs in 204 countries and territories in 2019. [file Table_2.DOCX]

Supplementary Table S2: ASPR, ASMR, and ASDR (per 100,000) of ILD, and SDIs in 204 countries and territories in 2019

| Location | ASPR | ASMR | ASDR | SDI | SDI level |
| --- | --- | --- | --- | --- | --- |
| Afghanistan | 32.85 | 1.19 | 27.05 | 0.343 | Low SDI |
| Albania | 54.15 | 0.78 | 21.1 | 0.681 | Middle SDI |
| Algeria | 37.87 | 1.02 | 23.39 | 0.652 | Middle SDI |
| American Samoa | 45.24 | 1.05 | 39.51 | 0.712 | High-middle SDI |
| Andorra | 51.37 | 2.76 | 55.26 | 0.894 | High SDI |
| Angola | 27.63 | 1.1 | 24.91 | 0.47 | Low-middle SDI |
| Antigua and Barbuda | 21.55 | 0.71 | 16.32 | 0.743 | High-middle SDI |
| Argentina | 80.74 | 3.48 | 72.07 | 0.708 | High-middle SDI |
| Armenia | 53.93 | 2.63 | 49.95 | 0.689 | Middle SDI |
| Australia | 65.23 | 2.54 | 48 | 0.839 | High SDI |
| Austria | 52.14 | 0.89 | 22.55 | 0.849 | High SDI |
| Azerbaijan | 31.34 | 1.19 | 27.23 | 0.683 | Middle SDI |
| Bahrain | 62.4 | 3.03 | 53.95 | 0.751 | High-middle SDI |
| Bangladesh | 63.53 | 2.84 | 63.95 | 0.483 | Low-middle SDI |
| Barbados | 28.19 | 1.37 | 31.09 | 0.742 | High-middle SDI |
| Belarus | 44.68 | 0.3 | 11.86 | 0.745 | High-middle SDI |
| Belgium | 48.64 | 1.56 | 33.28 | 0.851 | High SDI |
| Belize | 68.62 | 4.67 | 122.21 | 0.603 | Low-middle SDI |
| Benin | 21.87 | 0.57 | 15.02 | 0.352 | Low SDI |
| Bermuda | 63.25 | 3.35 | 75.28 | 0.813 | High SDI |
| Bhutan | 63.22 | 4.22 | 83.02 | 0.455 | Low-middle SDI |
| Bolivia | 90.28 | 11.53 | 194.36 | 0.566 | Low-middle SDI |
| Bosnia and Herzegovina | 49.37 | 0.55 | 16.57 | 0.718 | High-middle SDI |
| Botswana | 47.09 | 1.81 | 41.8 | 0.634 | Middle SDI |
| Brazil | 20.67 | 1.44 | 31.51 | 0.64 | Middle SDI |
| Brunei | 135.43 | 3.69 | 81.91 | 0.823 | High SDI |
| Bulgaria | 46.64 | 0.33 | 12.75 | 0.764 | High-middle SDI |
| Burkina Faso | 20.51 | 0.4 | 11.19 | 0.257 | Low SDI |
| Burundi | 21.94 | 1.12 | 26.8 | 0.284 | Low SDI |
| Cambodia | 14.35 | 0.64 | 13.86 | 0.469 | Low-middle SDI |
| Cameroon | 23.36 | 0.71 | 18.19 | 0.49 | Low-middle SDI |
| Canada | 116.47 | 3.01 | 65.1 | 0.873 | High SDI |
| Cape Verde | 21.95 | 0.36 | 10.31 | 0.525 | Low-middle SDI |
| Central African Republic | 24.2 | 1.72 | 37.93 | 0.274 | Low SDI |
| Chad | 21.55 | 0.75 | 18.73 | 0.238 | Low SDI |
| Chile | 141.67 | 6.43 | 121.49 | 0.759 | High-middle SDI |
| China | 35.62 | 0.45 | 12.36 | 0.686 | Middle SDI |
| Colombia | 33.65 | 0.93 | 25.55 | 0.633 | Middle SDI |
| Comoros | 22.51 | 0.93 | 22 | 0.455 | Low-middle SDI |
| Congo (Brazzaville) | 28.64 | 1.38 | 30.06 | 0.568 | Low-middle SDI |
| Cook Islands | 57.06 | 1.09 | 37.91 | 0.764 | High-middle SDI |
| Costa Rica | 70.25 | 3.1 | 70.31 | 0.68 | Middle SDI |
| Cote d'Ivoire | 20.94 | 0.53 | 13.79 | 0.408 | Low SDI |
| Croatia | 60.68 | 0.31 | 13.63 | 0.794 | High-middle SDI |
| Cuba | 18.44 | 0.34 | 11.36 | 0.668 | Middle SDI |
| Cyprus | 67.02 | 3.67 | 64.25 | 0.841 | High SDI |
| Czech Republic | 63.48 | 1.03 | 29.15 | 0.828 | High SDI |
| Denmark | 45.97 | 1.65 | 38.17 | 0.89 | High SDI |
| Djibouti | 24.23 | 0.92 | 22.1 | 0.459 | Low-middle SDI |
| Dominica | 25.16 | 1.31 | 30.49 | 0.729 | High-middle SDI |
| Dominican Republic | 18.18 | 0.61 | 16.86 | 0.592 | Low-middle SDI |
| DR Congo | 27.51 | 1.67 | 35.98 | 0.382 | Low SDI |
| Ecuador | 86.51 | 6.31 | 109.1 | 0.64 | Middle SDI |
| Egypt | 44.75 | 1.39 | 34.55 | 0.658 | Middle SDI |
| El Salvador | 46.73 | 1.94 | 42.44 | 0.573 | Low-middle SDI |
| Equatorial Guinea | 36.53 | 1.55 | 33.08 | 0.685 | Middle SDI |
| Eritrea | 23.16 | 1.03 | 25.1 | 0.396 | Low SDI |
| Estonia | 49.42 | 0.56 | 19.26 | 0.835 | High SDI |
| eSwatini | 40.72 | 1.74 | 39.61 | 0.577 | Low-middle SDI |
| Ethiopia | 19.74 | 0.87 | 20.09 | 0.343 | Low SDI |
| Federated States of Micronesia | 57.69 | 2.23 | 80.19 | 0.58 | Low-middle SDI |
| Fiji | 41.09 | 0.72 | 29.45 | 0.664 | Middle SDI |
| Finland | 59.51 | 2.05 | 40.6 | 0.856 | High SDI |
| France | 42.78 | 1.41 | 29.49 | 0.834 | High SDI |
| Gabon | 28.69 | 1.2 | 26.74 | 0.656 | Middle SDI |
| Georgia | 27.45 | 1.12 | 28.07 | 0.702 | High-middle SDI |
| Germany | 45.95 | 1.49 | 32.27 | 0.898 | High SDI |
| Ghana | 22.21 | 0.87 | 21.54 | 0.557 | Low-middle SDI |
| Greece | 38.42 | 1.31 | 28.21 | 0.794 | High-middle SDI |
| Greenland | 125.53 | 5.03 | 107.8 | 0.761 | High-middle SDI |
| Grenada | 28.96 | 1.31 | 32.39 | 0.669 | Middle SDI |
| Guam | 108.32 | 3.48 | 120.24 | 0.813 | High SDI |
| Guatemala | 44.48 | 2.38 | 52.45 | 0.526 | Low-middle SDI |
| Guinea | 21.33 | 0.61 | 15.85 | 0.355 | Low SDI |
| Guinea-Bissau | 20.79 | 0.64 | 16.94 | 0.325 | Low SDI |
| Guyana | 25.34 | 1.35 | 35.51 | 0.618 | Middle SDI |
| Haiti | 22.46 | 2.14 | 52.01 | 0.432 | Low SDI |
| Honduras | 46.8 | 3.88 | 87.23 | 0.496 | Low-middle SDI |
| Hungary | 58.3 | 0.9 | 26.44 | 0.791 | High-middle SDI |
| Iceland | 51.96 | 1.43 | 32.28 | 0.869 | High SDI |
| India | 73.6 | 5.75 | 116.62 | 0.566 | Low-middle SDI |
| Indonesia | 16.3 | 0.87 | 19.5 | 0.66 | Middle SDI |
| Iran | 33.17 | 0.57 | 14.74 | 0.67 | Middle SDI |
| Iraq | 36.41 | 0.85 | 21.38 | 0.671 | Middle SDI |
| Ireland | 81.5 | 3.69 | 71.24 | 0.867 | High SDI |
| Israel | 35.22 | 1.21 | 27.08 | 0.803 | High-middle SDI |
| Italy | 66.94 | 1.24 | 30.37 | 0.801 | High-middle SDI |
| Jamaica | 20.87 | 0.79 | 21.56 | 0.684 | Middle SDI |
| Japan | 162.61 | 3.11 | 68.61 | 0.87 | High SDI |
| Jordan | 71.45 | 2.94 | 61.97 | 0.731 | High-middle SDI |
| Kazakhstan | 30.98 | 1.12 | 29.23 | 0.723 | High-middle SDI |
| Kenya | 24.97 | 1.12 | 25.93 | 0.508 | Low-middle SDI |
| Kiribati | 43.06 | 1.21 | 46.62 | 0.527 | Low-middle SDI |
| Kuwait | 60.99 | 2.31 | 43.91 | 0.851 | High SDI |
| Kyrgyzstan | 27.38 | 0.3 | 9.22 | 0.596 | Low-middle SDI |
| Laos | 17.08 | 0.91 | 20.75 | 0.49 | Low-middle SDI |
| Latvia | 52.82 | 0.43 | 15.55 | 0.82 | High SDI |
| Lebanon | 42.79 | 0.87 | 21.43 | 0.708 | High-middle SDI |
| Lesotho | 38.21 | 1.86 | 41.04 | 0.507 | Low-middle SDI |
| Liberia | 20.4 | 0.36 | 10.24 | 0.37 | Low SDI |
| Libya | 38.45 | 1.08 | 25.52 | 0.709 | High-middle SDI |
| Lithuania | 38.23 | 0.31 | 11.61 | 0.843 | High SDI |
| Luxembourg | 43.15 | 1.26 | 28.12 | 0.895 | High SDI |
| Madagascar | 25.63 | 1.36 | 32.9 | 0.396 | Low SDI |
| Malawi | 22.42 | 0.89 | 21.59 | 0.384 | Low SDI |
| Malaysia | 23.91 | 1.12 | 25.28 | 0.737 | High-middle SDI |
| Maldives | 122.45 | 6.93 | 143.81 | 0.562 | Low-middle SDI |
| Mali | 24.54 | 0.81 | 21.62 | 0.263 | Low SDI |
| Malta | 75.89 | 2.77 | 58.84 | 0.801 | High-middle SDI |
| Marshall Islands | 57.84 | 2.2 | 81.5 | 0.544 | Low-middle SDI |
| Mauritania | 22.36 | 0.5 | 12.86 | 0.496 | Low-middle SDI |
| Mauritius | 87.75 | 5.55 | 107.33 | 0.705 | High-middle SDI |
| Mexico | 57.26 | 2.91 | 65.03 | 0.649 | Middle SDI |
| Moldova | 42.74 | 0.24 | 10.4 | 0.696 | High-middle SDI |
| Monaco | 49.8 | 1.71 | 36.6 | 0.902 | High SDI |
| Mongolia | 34.02 | 1.26 | 33.79 | 0.606 | Low-middle SDI |
| Montenegro | 47.6 | 0.25 | 10.32 | 0.791 | High-middle SDI |
| Morocco | 35.78 | 1.15 | 24.89 | 0.548 | Low-middle SDI |
| Mozambique | 21.96 | 0.75 | 18.5 | 0.307 | Low SDI |
| Myanmar | 31.21 | 1.56 | 33.66 | 0.521 | Low-middle SDI |
| Namibia | 42.96 | 2.02 | 43.99 | 0.612 | Middle SDI |
| Nauru | 53.52 | 2.36 | 93.06 | 0.618 | Middle SDI |
| Nepal | 63.64 | 10.09 | 195.14 | 0.422 | Low SDI |
| Netherlands | 48.95 | 1.55 | 32.95 | 0.883 | High SDI |
| New Zealand | 52.58 | 2.04 | 39.55 | 0.84 | High SDI |
| Nicaragua | 32.85 | 1.56 | 33.09 | 0.517 | Low-middle SDI |
| Niger | 22.22 | 0.81 | 20.13 | 0.162 | Low SDI |
| Nigeria | 22.1 | 0.66 | 15.86 | 0.515 | Low-middle SDI |
| Niue | 56.82 | 1.73 | 68.86 | 0.711 | High-middle SDI |
| North Korea | 23.6 | 0.52 | 14.06 | 0.558 | Low-middle SDI |
| North Macedonia | 46.67 | 0.27 | 11.05 | 0.744 | High-middle SDI |
| Northern Mariana Islands | 97.7 | 3.09 | 102.35 | 0.771 | High-middle SDI |
| Norway | 84.36 | 1.72 | 40.26 | 0.913 | High SDI |
| Oman | 40.43 | 1.23 | 27.82 | 0.783 | High-middle SDI |
| Pakistan | 33.05 | 3.33 | 69.05 | 0.449 | Low SDI |
| Palau | 123.9 | 3.85 | 131.13 | 0.738 | High-middle SDI |
| Palestine | 97.73 | 4.5 | 97.52 | 0.588 | Low-middle SDI |
| Panama | 53.8 | 2.01 | 48 | 0.686 | Middle SDI |
| Papua New Guinea | 96.83 | 3.19 | 110.6 | 0.394 | Low SDI |
| Paraguay | 20.09 | 1.06 | 23.56 | 0.638 | Middle SDI |
| Peru | 150.54 | 10.78 | 190.13 | 0.648 | Middle SDI |
| Philippines | 10.54 | 0.26 | 6.78 | 0.623 | Middle SDI |
| Poland | 54.4 | 0.67 | 21.54 | 0.802 | High-middle SDI |
| Portugal | 48.24 | 2.07 | 44.1 | 0.743 | High-middle SDI |
| Puerto Rico | 45.12 | 2.2 | 51.07 | 0.814 | High SDI |
| Qatar | 39.21 | 0.79 | 17.49 | 0.83 | High SDI |
| Romania | 64.22 | 1.39 | 37.97 | 0.76 | High-middle SDI |
| Russia | 39.96 | 0.28 | 10.92 | 0.805 | High-middle SDI |
| Rwanda | 25.38 | 1.13 | 27.28 | 0.429 | Low SDI |
| Saint Kitts and Nevis | 31.18 | 1.76 | 39.58 | 0.746 | High-middle SDI |
| Saint Lucia | 35.4 | 1.95 | 49.2 | 0.67 | Middle SDI |
| Saint Vincent and the Grenadines | 22.46 | 1.02 | 25.43 | 0.627 | Middle SDI |
| Samoa | 55.21 | 1.94 | 67.97 | 0.641 | Middle SDI |
| San Marino | 26.39 | 0.46 | 11.37 | 0.884 | High SDI |
| Sao Tome and Principe | 31.53 | 1.47 | 34.69 | 0.502 | Low-middle SDI |
| Saudi Arabia | 114.44 | 5.67 | 105.62 | 0.805 | High-middle SDI |
| Senegal | 22 | 0.62 | 15.71 | 0.389 | Low SDI |
| Serbia | 41.47 | 0.47 | 15.12 | 0.767 | High-middle SDI |
| Seychelles | 15.67 | 0.58 | 13.51 | 0.724 | High-middle SDI |
| Sierra Leone | 20.61 | 0.52 | 13.89 | 0.347 | Low SDI |
| Singapore | 109.1 | 0.77 | 27.05 | 0.861 | High SDI |
| Slovakia | 55.16 | 0.7 | 23.22 | 0.812 | High SDI |
| Slovenia | 64.92 | 0.84 | 22.65 | 0.84 | High SDI |
| Solomon Islands | 48.43 | 2.26 | 73.85 | 0.407 | Low SDI |
| Somalia | 20.73 | 1.09 | 26.11 | 0.081 | Low SDI |
| South Africa | 53.23 | 2.2 | 47.62 | 0.678 | Middle SDI |
| South Korea | 102.19 | 1.87 | 43.07 | 0.878 | High SDI |
| South Sudan | 22.95 | 1.05 | 23.9 | 0.363 | Low SDI |
| Spain | 73.34 | 3.16 | 59.67 | 0.767 | High-middle SDI |
| Sri Lanka | 20.11 | 0.69 | 16.48 | 0.69 | High-middle SDI |
| Sudan | 36.04 | 1.36 | 30.69 | 0.515 | Low-middle SDI |
| Suriname | 26.49 | 1.45 | 37.46 | 0.636 | Middle SDI |
| Sweden | 59.69 | 2.01 | 40.82 | 0.872 | High SDI |
| Switzerland | 44.15 | 1.46 | 30.46 | 0.929 | High SDI |
| Syria | 44.62 | 1.48 | 33.21 | 0.619 | Middle SDI |
| Taiwan (province of China) | 30.65 | 0.73 | 17.29 | 0.868 | High SDI |
| Tajikistan | 64.2 | 4.18 | 84.09 | 0.539 | Low-middle SDI |
| Tanzania | 22.56 | 0.82 | 20.25 | 0.423 | Low SDI |
| Thailand | 13.55 | 0.33 | 9.18 | 0.687 | Middle SDI |
| The Bahamas | 34.08 | 1.84 | 44.9 | 0.796 | High-middle SDI |
| The Gambia | 21.33 | 0.64 | 16.24 | 0.399 | Low SDI |
| Timor-Leste | 16.13 | 0.96 | 21.11 | 0.514 | Low-middle SDI |
| Togo | 21.48 | 0.56 | 14.96 | 0.417 | Low SDI |
| Tokelau | 50.08 | 1.73 | 61.8 | 0.626 | Middle SDI |
| Tonga | 52.63 | 1.38 | 50.81 | 0.636 | Middle SDI |
| Trinidad and Tobago | 32.82 | 1.67 | 43.23 | 0.757 | High-middle SDI |
| Tunisia | 39.78 | 0.91 | 22.24 | 0.672 | Middle SDI |
| Turkey | 50.91 | 1.17 | 30.11 | 0.748 | High-middle SDI |
| Turkmenistan | 28.24 | 0.45 | 13.93 | 0.67 | Middle SDI |
| Tuvalu | 48.96 | 2.05 | 74.33 | 0.589 | Low-middle SDI |
| Uganda | 25.43 | 1.24 | 29.21 | 0.404 | Low SDI |
| UK | 87.01 | 3.99 | 76.69 | 0.847 | High SDI |
| Ukraine | 37.55 | 0.5 | 17.2 | 0.736 | High-middle SDI |
| United Arab Emirates | 44.34 | 1.49 | 41.69 | 0.88 | High SDI |
| Uruguay | 62.46 | 2.28 | 48.6 | 0.697 | High-middle SDI |
| USA | 126 | 3.66 | 79.87 | 0.859 | High SDI |
| Uzbekistan | 44.3 | 2.06 | 45.48 | 0.631 | Middle SDI |
| Vanuatu | 48.72 | 2.36 | 87.72 | 0.485 | Low-middle SDI |
| Venezuela | 34.51 | 1.37 | 31.86 | 0.607 | Low-middle SDI |
| Vietnam | 19.69 | 0.76 | 16.37 | 0.617 | Middle SDI |
| Virgin Islands | 45.1 | 2.56 | 58.02 | 0.799 | High-middle SDI |
| Yemen | 33.48 | 1.13 | 25.91 | 0.412 | Low SDI |
| Zambia | 24.02 | 1.03 | 24.99 | 0.505 | Low-middle SDI |
| Zimbabwe | 33.16 | 0.67 | 17.39 | 0.476 | Low-middle SDI |
