# Supplementary material for: Global trends of interstitial lung diseases from 1990 to 2019: an age–period–cohort study based on the Global Burden of Disease study 2019, and projections until 2030
Source: Front Med (Lausanne). 2023 Jul 24;10:1141372. doi: 10.3389/fmed.2023.1141372 (PMC10404716; doi:10.3389/fmed.2023.1141372)
Supplement: Supplementary Table 3 — Joinpoint regression analysis for global trends of ASPR, ASMR, and ASDR (per 100,000) of ILD among both sexes, males, and females from 1990 to 2019. [file Table_3.DOCX]

Supplementary Table S3: Joinpoint regression analysis for global trends of ASPR, ASMR, and ASDR (per 100,000) of ILD among both sexes, males, and females from 1990 to 2019

| ASPR | | | |
| --- | --- | --- | --- |
| Sex | Period | APC (95% CI) | AAPC (95%CI) |
| Both | 1990-1994 | -0.8 (-1.13, -0.48) | 0.32 (0.19, 0.46) |
|  | 1994-2006 | 0.5 (0.43, 0.56) |  |
|  | 2006-2010 | 0.76 (0.33, 1.19) |  |
|  | 2010-2014 | -0.01 (-0.42, 0.41) # |  |
|  | 2014-2017 | 1.15 (0.28, 2.02) |  |
|  | 2017-2019 | 0.12 (-0.83, 1.09) # |  |
| Male | 1990-1994 | -0.87 (-1.19, -0.55) | 0.36 (0.23, 0.49) |
|  | 1994-2006 | 0.53 (0.47, 0.59) |  |
|  | 2006-2010 | 0.98 (0.56, 1.39) |  |
|  | 2010-2014 | 0.05 (-0.36, 0.45) # |  |
|  | 2014-2017 | 1.1 (0.26, 1.96) |  |
|  | 2017-2019 | 0.1 (-0.83, 1.03) # |  |
| Female | 1990-1994 | -0.78 (-1.26, -0.29) | 0.31 (0.19, 0.42) |
|  | 1994-2010 | 0.48 (0.42, 0.54) |  |
|  | 2010-2014 | 0.05 (-0.56, 0.67) # |  |
|  | 2014-2019 | 0.84 (0.53, 1.15) |  |
|  |  |  |  |
| ASMR | | | |
| Sex | Period | APC (95% CI) | AAPC (95%CI) |
| Both | 1990-1992 | 0.35 (-1.13, 1.85) # | 0.73 (0.62, 0.84) |
|  | 1992-2010 | 1.28 (1.23, 1.34) |  |
|  | 2010-2019 | -0.3 (-0.45, -0.14) |  |
| Male | 1990-2011 | 0.95 (0.91, 0.99) | 0.58 (0.52, 0.64) |
|  | 2011-2019 | -0.38 (-0.57, -0.18) |  |
| Female | 1990-1992 | 0.49 (-2.01, 3.06) # | 0.94 (0.76, 1.12) |
|  | 1992-2008 | 1.69 (1.59, 1.78) |  |
|  | 2008-2019 | -0.06 (-0.21, 0.1) # |  |
|  |  |  |  |
| ASDR | | | |
| Sex | Period | APC (95% CI) | AAPC (95%CI) |
| Both | 1990-2010 | 0.75 (0.69, 0.8) | 0.41 (0.35, 0.47) |
|  | 2010-2019 | -0.33 (-0.5, -0.15) |  |
| Male | 1990-2011 | 0.51 (0.46, 0.55) | 0.24 (0.19, 0.3) |
|  | 2011-2019 | -0.45 (-0.63, -0.27) |  |
| Female | 1990-2008 | 1.07 (0.99, 1.15) | 0.62 (0.54, 0.7) |
|  | 2008-2019 | -0.11 (-0.27, 0.05) # |  |

“#” indicates that the APC is not statistically significant from zero at the α = 0.05 level

AAPC, average annual percent change; APC, annual percent change; CI, confidence interval
