# Supplementary material for: Global trends of interstitial lung diseases from 1990 to 2019: an age–period–cohort study based on the Global Burden of Disease study 2019, and projections until 2030
Source: Front Med (Lausanne). 2023 Jul 24;10:1141372. doi: 10.3389/fmed.2023.1141372 (PMC10404716; doi:10.3389/fmed.2023.1141372)
Supplement: Supplementary Table 4 — Local drifts and net drifts for global trends of ASPR, ASMR, and ASDR (per 100,000) of ILD among both sexes, males, and females from 1990 to 2019. [file Table_4.DOCX]

Supplementary Table S4: local drifts and net drifts for global trends of ASPR, ASMR, and ASDR (per 100,000) of ILD among both sexes, males, and females from 1990 to 2019

| ASPR | | | | | | | | | | | | | |
| --- | --- | --- | --- | --- | --- | --- | --- | --- | --- | --- | --- | --- | --- |
| Local drift | | | | | | | | | | | | | |
| Age | Both | | Male | | Female |  | | Age | Both | | Male | | Female |
| <5 | 0.3191 | | 0.2629 | | 0.3852 |  | | 50-54 | -0.1333 | | -0.1757 | | -0.0943 |
| 5-9 | 0.262 | | 0.167 | | 0.3657 |  | | 55-59 | 0.1111 | | 0.0635 | | 0.1594 |
| 10-14 | 0.2149 | | 0.0932 | | 0.341 |  | | 60-64 | 0.3185 | | 0.2786 | | 0.3534 |
| 15-19 | 0.2598 | | 0.132 | | 0.3956 |  | | 65-69 | 0.5913 | | 0.5547 | | 0.61 |
| 20-24 | 0.3228 | | 0.2267 | | 0.4279 |  | | 70-74 | 0.8547 | | 0.8474 | | 0.8235 |
| 25-29 | 0.3042 | | 0.2546 | | 0.3595 |  | | 75-79 | 1.1773 | | 1.194 | | 1.1024 |
| 30-34 | 0.1881 | | 0.1811 | | 0.1926 |  | | 80-84 | 1.6906 | | 1.7236 | | 1.5847 |
| 35-39 | 0.0457 | | 0.0544 | | 0.0285 |  | | 85-89 | 2.216 | | 2.3337 | | 2.0575 |
| 40-44 | -0.1671 | | -0.1742 | | -0.1825 |  | | 90-94 | 2.6372 | | 2.8475 | | 2.4507 |
| 45-49 | -0.2679 | | -0.2924 | | -0.2585 |  | | 95+ | 3.0177 | | 3.1821 | | 2.8706 |
| Net drift | | | | | | | | | | | | | |
| Both | | 0.4711 | | Male | | | 0.4505 | | | Female | | 0.4796 | |

| ASMR | | | | | | | | | | | | | |
| --- | --- | --- | --- | --- | --- | --- | --- | --- | --- | --- | --- | --- | --- |
| Local drift | | | | | | | | | | | | | |
| Age | Both | | Male | | Female |  | | Age | Both | | Male | | Female |
| <5 | -0.4704 | | -1.0383 | | 0.0866 |  | | 50-54 | -0.2577 | | -0.2762 | | 0.0617 |
| 5-9 | -0.358 | | -0.822 | | 0.1744 |  | | 55-59 | -0.1233 | | -0.1528 | | 0.2185 |
| 10-14 | -0.2647 | | -0.5626 | | 0.1846 |  | | 60-64 | -0.1023 | | -0.1861 | | 0.3207 |
| 15-19 | -0.0659 | | -0.1918 | | 0.336 |  | | 65-69 | 0.0111 | | -0.17 | | 0.4879 |
| 20-24 | 0.1655 | | 0.1774 | | 0.5472 |  | | 70-74 | 0.0524 | | -0.1761 | | 0.5076 |
| 25-29 | 0.2761 | | 0.3067 | | 0.6336 |  | | 75-79 | -0.0261 | | -0.2524 | | 0.3703 |
| 30-34 | 0.1842 | | 0.1673 | | 0.5527 |  | | 80-84 | 0.054 | | -0.149 | | 0.3062 |
| 35-39 | 0.1205 | | 0.0759 | | 0.473 |  | | 85-89 | 0.2824 | | 0.1359 | | 0.2329 |
| 40-44 | -0.0358 | | -0.0983 | | 0.2519 |  | | 90-94 | 0.4775 | | 0.2668 | | 0.2745 |
| 45-49 | -0.2567 | | -0.2977 | | 0.027 |  | | 95+ | 0.6491 | | 0.0656 | | 0.5841 |
| Net drift | | | | | | | | | | | | | |
| Both | | -0.0026 | | Male | | | -0.1227 | | | Female | | 0.3365 | |

| ASDR | | | | | | | | | | | | | |
| --- | --- | --- | --- | --- | --- | --- | --- | --- | --- | --- | --- | --- | --- |
| Local drift | | | | | | | | | | | | | |
| Age | Both | | Male | | Female |  | | Age | Both | | Male | | Female |
| <5 | -0.4307 | | -0.8443 | | 0.0527 |  | | 50-54 | -0.2199 | | -0.2735 | | 0.0297 |
| 5-9 | -0.3099 | | -0.6633 | | 0.1183 |  | | 55-59 | -0.0833 | | -0.1396 | | 0.2011 |
| 10-14 | -0.2274 | | -0.4787 | | 0.1368 |  | | 60-64 | -0.0425 | | -0.156 | | 0.303 |
| 15-19 | -0.0346 | | -0.1743 | | 0.262 |  | | 65-69 | 0.077 | | -0.1142 | | 0.489 |
| 20-24 | 0.1876 | | 0.153 | | 0.4446 |  | | 70-74 | 0.1285 | | -0.0747 | | 0.5292 |
| 25-29 | 0.2839 | | 0.2818 | | 0.5094 |  | | 75-79 | 0.0906 | | -0.0984 | | 0.4265 |
| 30-34 | 0.1692 | | 0.1594 | | 0.4093 |  | | 80-84 | 0.2087 | | 0.0571 | | 0.452 |
| 35-39 | 0.0915 | | 0.0602 | | 0.3361 |  | | 85-89 | 0.4693 | | 0.3779 | | 0.4495 |
| 40-44 | -0.064 | | -0.1193 | | 0.1485 |  | | 90-94 | 0.6805 | | 0.5499 | | 0.5285 |
| 45-49 | -0.2423 | | -0.2939 | | -0.0346 |  | | 95+ | 0.8394 | | 0.3466 | | 0.808 |
| Net drift | | | | | | | | | | | | | |
| Both | | 0.0443 | | Male | | | -0.0688 | | | Female | | 0.3098 | |
