# Supplementary material for: Global trends of interstitial lung diseases from 1990 to 2019: an age–period–cohort study based on the Global Burden of Disease study 2019, and projections until 2030
Source: Front Med (Lausanne). 2023 Jul 24;10:1141372. doi: 10.3389/fmed.2023.1141372 (PMC10404716; doi:10.3389/fmed.2023.1141372)
Supplement: Supplementary Table 5 — Wald Chi-Square tests for estimable functions in the APC model. [file Table_5.DOCX]

Supplementary Table S5: Wald Chi-Square tests for estimable functions in the APC model

| ASPR | | | | | | |
| --- | --- | --- | --- | --- | --- | --- |
|  | Both | P Value | Male | P Value | Female | P Value |
| NetDrift = 0 | 395.1 | <0.001 | 487.5 | <0.001 | 297.8 | <0.001 |
| All Age Deviations = 0 | 13136.3 | <0.001 | 13808.6 | <0.001 | 11543.2 | <0.001 |
| All Period Deviations = 0 | 69.0 | <0.001 | 123.9 | <0.001 | 34.7 | <0.001 |
| All Cohort Deviations = 0 | 1488.7 | <0.001 | 2109.3 | <0.001 | 1003.5 | <0.001 |
| All Period RR = 1 | 496.6 | <0.001 | 662.6 | <0.001 | 349.2 | <0.001 |
| All Cohort RR = 1 | 2481.1 | <0.001 | 3334.9 | <0.001 | 1744.5 | <0.001 |
| All Local Drifts = Net Drift | 1484.8 | <0.001 | 2103.3 | <0.001 | 1000.1 | <0.001 |

| ASMR | | | | | | |
| --- | --- | --- | --- | --- | --- | --- |
|  | Both | P Value | Male | P Value | Female | P Value |
| NetDrift = 0 | 0.0 | 0.947 | 5.0 | 0.025 | 28.6 | <0.001 |
| All Age Deviations = 0 | 11811.4 | <0.001 | 4618.4 | <0.001 | 4668.7 | <0.001 |
| All Period Deviations = 0 | 87.3 | <0.001 | 33.3 | <0.001 | 32.6 | <0.001 |
| All Cohort Deviations = 0 | 162.0 | <0.001 | 84.6 | <0.001 | 58.3 | <0.001 |
| All Period RR = 1 | 89.1 | <0.001 | 41.5 | <0.001 | 53.7 | <0.001 |
| All Cohort RR = 1 | 163.3 | <0.001 | 120.0 | <0.001 | 209.4 | <0.001 |
| All Local Drifts = Net Drift | 151.8 | <0.001 | 64.6 | <0.001 | 58.0 | <0.001 |

| ASDR | | | | | | |
| --- | --- | --- | --- | --- | --- | --- |
|  | Both | P Value | Male | P Value | Female | P Value |
| NetDrift = 0 | 8.4 | 0.004 | 9.8 | 0.002 | 285.9 | <0.001 |
| All Age Deviations = 0 | 48801.9 | <0.001 | 19360.9 | <0.001 | 33613.3 | <0.001 |
| All Period Deviations = 0 | 138.4 | <0.001 | 32.1 | <0.001 | 100.7 | <0.001 |
| All Cohort Deviations = 0 | 461.9 | <0.001 | 239.9 | <0.001 | 350.5 | <0.001 |
| All Period RR = 1 | 139.9 | <0.001 | 46.7 | <0.001 | 345.2 | <0.001 |
| All Cohort RR = 1 | 472.3 | <0.001 | 281.6 | <0.001 | 1018.6 | <0.001 |
| All Local Drifts = Net Drift | 452.1 | <0.001 | 212.9 | <0.001 | 345.2 | <0.001 |
